# A kinetic model of iron trafficking in growing *Saccharomyces cerevisiae* cells; applying mathematical methods to minimize the problem of sparse data and generate viable autoregulatory mechanisms

**DOI:** 10.1371/journal.pcbi.1011701

**Published:** 2023-12-19

**Authors:** Shantanu Thorat, Jay R. Walton, Paul A. Lindahl

**Affiliations:** 1 Department of Computer Science and Engineering, Texas A&M University, College Station, Texas, United States of America; 2 Department of Mathematics, Texas A&M University, College Station Texas, Texas, United States of America; 3 Department of Chemistry, Texas A&M University, College Station Texas, Texas, United States of America; 4 Department of Biochemistry and Biophysics, Texas A&M University, College Station Texas, Texas, United States of America; University of California Riverside, UNITED STATES

## Abstract

Iron is an essential transition metal for all eukaryotic cells, and its trafficking throughout the cell is highly regulated. However, the overall cellular mechanism of regulation is poorly understood despite knowing many of the molecular players involved. Here, an ordinary-differential-equations (ODE) based kinetic model of iron trafficking within a growing yeast cell was developed that included autoregulation. The 9-reaction 8-component *in-silico* cell model was solved under both steady-state and time-dependent dynamical conditions. The ODE for each component included a dilution term due to cell growth. Conserved rate relationships were obtained from the null space of the stoichiometric matrix, and the reduced-row-echelon-form was used to distinguish independent from dependent rates. Independent rates were determined from experimentally estimated component concentrations, cell growth rates, and the literature. Simple rate-law expressions were assumed, allowing rate-constants for each reaction to be estimated. Continuous Heaviside logistical functions were used to regulate rate-constants. These functions acted like valves, opening or closing depending on component “sensor” concentrations. Two cellular regulatory mechanisms were selected from 134,217,728 possibilities using a novel approach involving 6 mathematically-defined filters. Three cellular states were analyzed including healthy wild-type cells, iron-deficient wild-type cells, and a frataxin-deficient strain of cells characterizing the disease Friedreich’s Ataxia. The model was stable toward limited perturbations, as determined by the eigenvalues of Jacobian matrices. Autoregulation allowed healthy cells to transition to the diseased state when triggered by a mutation in frataxin, and to the iron-deficient state when cells are placed in iron-deficient growth medium. The *in-silico* phenotypes observed during these transitions were similar to those observed experimentally. The model also predicted the observed effects of hypoxia on the diseased condition. A similar approach could be used to solve ODE-based kinetic models associated with other biochemical processes operating within growing cells.

## Author summary

Developing mathematical models that describe the rates of biochemical processes within growing cells can be difficult because sufficient experimental information is often unavailable. This is especially true for models that include regulatory mechanisms. Here we developed a mathematical model describing the kinetics of how iron, an essential transition metal, is “trafficked” into and within a eukaryotic cell, and how such a system *might* be regulated. The model was solved using a novel combination of mathematical methods combined with a modest amount of experimental data. Once solved, the model simulated how iron trafficking is affected when healthy cells become diseased or iron deficient. It also accurately predicted the healing effect of reducing the level of oxygen to which diseased cells are exposed. Models of other biochemical processes occurring within growing cells could be developed using similar methods. Such models may eventually be useful for developing more effective treatments for metabolic diseases involving complex biochemical reaction networks.

## Introduction

Nearly all biochemical processes occurring within cells are regulated, often at multiple levels. The molecular players involved in regulation are typically identified by genetic screens in which the deletion of a particular gene displays a dysregulated phenotype. However, understanding how these players collectively interact to give rise to such phenotypes remains a challenge [1, 2]. Various approaches to modeling the regulation of biochemical processes in cells have been reviewed [3]; our focus here is on building a cellular-level (a.k.a. systems’ level) regulatory mechanism within a classical deterministic ordinary-differential-equations- (ODE-) based biochemical reaction network [4, 5]. Specifically, we wanted to develop a cellular-level iron trafficking network, including autoregulation, for budding yeast *Saccharomyces cerevisiae*.

Iron is essential in biology, and the import and function of this transition metal has been the subject of many previous computational studies [6–11]. In the current model, we wanted to simulate observed iron-associated changes in healthy iron-replete wild-type (WT) cells as they transitioned to either an iron-deficient state or to a diseased state in which expression of the yeast frataxin homolog 1 (Yfh1) protein is anomalously low. Yfh1 is a mitochondrial protein involved in the synthesis of iron-sulfur clusters (ISCs) and possibly of heme centers. The primary biochemical role of Yfh1 is to activate cysteine desulfurase, the enzyme responsible for extracting sulfur from cysteine–the sulfur that is ultimately used to build [Fe_2_S_2_] and [Fe_4_S_4_] clusters in mitochondria [12]. Humans with a deficiency of frataxin, the human homolog of Yfh1, suffer from Friedreich’s Ataxia, a neurodegenerative mitochondrial disease that involves iron dysregulation [13]. We wanted to understand the mechanism of disease progression with hopes that this might help in developing more effective treatments. The observed changes in phenotype between healthy and diseased human *vs*. yeast cells are similar, in that a deficiency of frataxin in humans and of Yfh1 in yeast both result in a massive accumulation of nanoparticle iron in mitochondria, a decline in iron-sulfur clusters, an increase in ROS, and the dysregulation of iron trafficking [14].

Developing reliable ODE-based models of biochemical processes within cells is often challenging because the actual mechanisms are rarely known in sufficient detail. Moreover, a complete set of experimentally determined kinetic parameters, as is required to numerically integrate an ODE system, including rate-constants (*k*_*rxn*_), Michaelis-Menten (*K*_*m*_) constants, and component concentrations [C_i_], are rarely known, or known with sufficient accuracy. Both problems have been considered for decades and methods are well established [15–17].

For the model developed here, the problem of insufficient data was handled in the standard way; by grouping molecular species, combining reactions into fewer more symbolic forms, and assuming parsimonious rate-law expressions. Resulting *coarse-grain* models have fewer unknown parameters and simpler reaction networks; thus, they are more readily solved mathematically. However, the symbolic nature of reactions and components limits their testability and connection to reality. Nevertheless, even “sloppy” models, in which some parameters are guessed, can provide useful insights [18].

**Fig 1:**
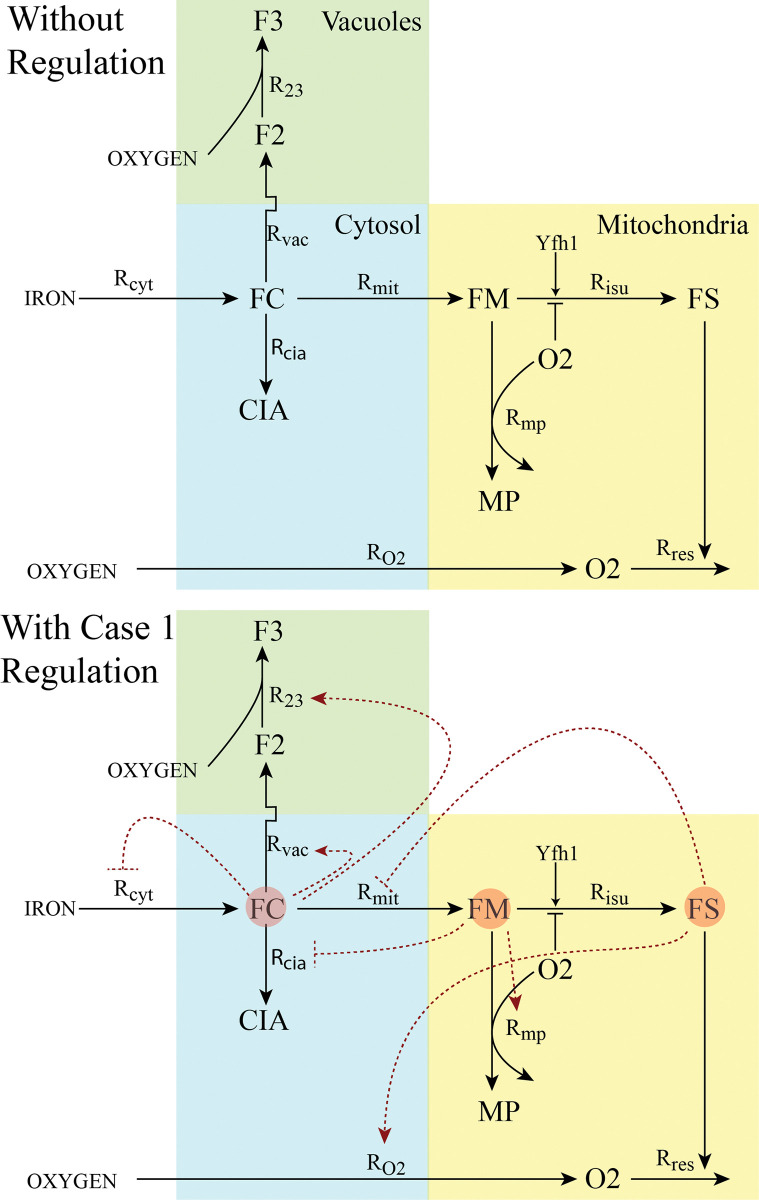
The chemical model. Top, without regulation; blue, yellow, and green regions represent cytosol, mitochondria, and vacuoles, respectively. Nutrient IRON enters the cytosol and becomes part of the labile iron pool (FC). Iron from this pool can either enter mitochondria, vacuole, or remain in the cytosol, converting to the CIA. This component was named after the Cytosolic Iron-sulfur Assembly protein complex, which functions to assemble iron-sulfur clusters in the cytosol. In this model, the CIA refers to all iron from FC that is not imported into mitochondria or vacuoles. Iron that enters vacuoles is stored, either as Fe^II^ (F2) or Fe^III^ (F3). Iron that enters mitochondria becomes part of another labile iron pool called FM which is used as substrate for the assembly of iron-sulfur clusters and hemes (FS). These centers are installed into, among other proteins, the respiratory complexes which ultimately reduce O2 to water. In healthy cells, this prevents O2 from penetrating into the mitochondrial matrix. However, in the diseased state, the “Respiratory shield” is weakened, and excessive O2 enters the matrix and reacts with FM to generate nanoparticles (MP) and reactive oxygen species (ROS). ROS is not shown as it is not included in the model (however, its rate of formation would be the same as MP). Names of reaction rates are indicated near the associated arrow. Bottom Panel: with regulation (CRM Case 1) shown in red dashed lines. Circles indicate sensed species. Feedback terms are indicated by perpendicular terminal lines. Feedforward terms are indicated by terminal arrows. According to this regulatory mechanism, there are three sensed species, including FC, FM, and FS. FC controls the rate of iron import into the cell, the rate of cytosolic iron import in vacuoles, and the oxidation of vacuolar Fe^II^ to Fe^III^. FM controls the rate at which the cytosolic labile iron pool reacts to form the CIA, and the rate at which FM reactions with O2 to make nanoparticles. FS controls the rate by which cytosolic iron enters mitochondria and the rate by which nutrient OXYGEN enters the cell.

Here we utilized the 9-reaction, 8-component coarse-grain model shown in Fig 1 (top panel), which was developed from earlier versions [19, 20]. The assumed chemical mechanism was translated into a set of 8 ODEs, one for each component *C*_*i*_. Each ODE included rate-law terms that influence [C_i_], the concentration of *C*_*i*_. These influences included substrate dependences controlled by either Michaelis-Menten or mass-action expressions. In each rate, the expression assumed depended on whether the reaction was, or was not, presumed to be enzyme-catalyzed. Each ODE also included a term that accounted for the dilution of each component due to cell growth. This term had the form -*α*_*cell*_ [C_i_] where *α*_*cell*_ is the exponential growth rate of the cell, measured experimentally as the slope of ln(absorbance at 600 nm) vs. time for a culture of growing cells [21]. (A_600_ is proportional to the extent of light scattering, and thus to cell density in cultures.) Concentrations of iron-containing components in the model were estimated from published Mössbauer spectra and the iron content of whole yeast cells, and of isolated mitochondria, cytosol, and vacuoles [20–27]. The model assumed 3 cellular compartments, including cytosol, mitochondria, and vacuoles. Modeling within the context of cellular compartments is well-established [19, 28].

Three cellular states were used to train the model, including W (WT cells grown on iron-replete media), Y (the Yfh1-deficient strain), and D (WT cells grown on iron-deficient media). Each cellular state employed the same biochemical reaction network but different rates of reactions. The combined effect of these rates controlled component concentrations. Reaction rates could be affected by external factors such as [IRON] and [OXYGEN] in the growth media, and by internal factors which included, in this case, the mutation of the *YFH1* gene and the growth rate of the cells.

The mechanisms used to regulate reaction rates in real cells are often complicated and/or poorly understood. This is especially true in assessing how individual regulatory processes function in concert at the systems’ (i.e. cellular) level. Methods for optimizing regulatory mechanisms in biological systems have also been investigated [15, 17]. Here we used *surrogate* regulatory mechanisms that were simpler than actual chemical mechanisms but still able to mimic the behavior arising from the actual mechanisms. We wanted these regulatory mechanisms to cause cells to transition from one cellular state to another (W → Y or W → D) as realistically as possible. The selection process that distinguished realistic from unrealistic behavior was analogous to evolutionary selection. In our case, this involved mathematically screening candidate mechanisms and selecting those which afforded transitions most likely to be observed in real cells.

In real yeast cells, the best-understood regulatory mechanism associated with iron trafficking involves the *iron regulon*, 20–30 genes whose expression levels are controlled by proteins Aft1 and Aft2. These two transcription factors can exist in metal-free (apo-) forms which bind promotor sequences of iron-regulon genes; binding promotes gene expression. Aft1/2 can also exist in an inactive holo-form in which an [Fe_2_S_2_] cluster bridges Aft1 or Aft2 homodimers. The cellular functions of Aft1 *vs*. Aft2 differ slightly [29] but these differences were ignored here. The balance between apo- and holo- forms is controlled by the extent of ISC activity occurring in mitochondria. When ISC activity is normal (WT cells growing under iron-replete conditions), the holo forms of Aft1/2 dominate and expression of the iron regulon declines. When the apo- forms dominate (under iron-deficient conditions), the iron regulon activates. Activation causes additional iron to be imported into the cell and funneled into the mitochondria to stimulate ISC assembly.

To some extent, Aft1/2 also control the level of iron imported into vacuoles for iron storage. Prior to ca. 2004, cellular iron regulation was thought to be controlled (or sensed) by the labile iron pool in the cytosol [30–32]. However, Chen et al. found that mitochondrial ISC activity rather than this pool was being sensed [33]. Complicating matters is that proteins Yap5 and Cth1/2 also regulate iron trafficking in yeast cells [34, 35]. In summary, there are numerous molecular systems involved in regulating iron trafficking and metabolism in yeast cells, but there are also major uncertainties regarding how these local mechanisms are integrated at the global or cellular level. Our objective was to better understand this integration, by using a fully transparent quantitative method to select the cellular-level surrogate-based regulatory mechanism(s) that could mimic the overall regulatory behavior of the cell.

Cellular regulation plays an essential role in generating the phenotype of Yfh1-deficient cells. Yfh1 deficiency causes iron dysregulation. Nutrient iron from the environment rushes into the cell, migrates through the cytosol, and traffics into mitochondria. Iron is simultaneously exported from vacuoles into the cytosol [36]. Iron in the mitochondrial matrix, which would otherwise be used as substrate for ISC and heme biosynthesis, reacts to form ferric phosphate oxyhydroxide nanoparticles. Associated with this is a decline in ISC assembly and heme biosynthesis, and an increase in reactive oxygen species which damages various cellular components.

Wofford and Lindahl, and more recently Fernandez et al., developed a core biochemical mechanism that explains this phenotype on the molecular level [19, 20]. Decisions regarding how best to regulate iron in those models were made at the discretion of the modeler. The current study was motivated to develop user-independent *autoregulatory* mechanisms (“auto” means that the system would be regulated exclusively and automatically by components in the system). Our success represents an advance not only for the field of cellular iron regulation, but for designing cellular-level autoregulatory mechanisms generally.

## Model development

### Biochemical reaction network

The mechanism of Fig 1 top panel defines the biochemical reaction network assumed for iron trafficking in yeast cells, *excluding regulation*. Reactions and rate-law expressions are given in Table 1.

**Table 1:** Chemical reactions, rate-law expressions, and cellular regions in which the reactions of Fig 1 occur. Regulation is not included. Regions: E, environment; C, cytosol; M, mitochondria; V, vacuole.

#	Reaction	Rate Law Expression	Region
1	IRON → FC	$R_{cyt}=\frac{k_{cyt}[IRON]}{K_{Cyt(IRON)}^{}+[IRON]}$	E, C
2	FC → FM	$R_{mit}=\frac{k_{mit}[FC]}{K_{mit(FC)}+[FC]}$	C, M
3	FC → F2	$R_{vac}=\frac{k_{vac}[FC]}{K_{vac(FC)}^{}+[FC]}$	C, V
4	FC → CIA	$R_{cia}=\frac{k_{cia}[FC]}{K_{cia(FC)}^{}+[FC]}$	C
5	FM → FS	$R_{isu}=\frac{k_{isu}[FM]}{K_{isu(FM)}^{}+[FM]}(\frac{{\left[O2\right]}_{sp}}{{\left[O2\right]}_{sp}+[O2]})$	M
6	FM + O2 → MP	$R_{mp}=k_{mp}[FM][O2]$	M
7	OXYGEN → O2	$R_{O2}=k_{O2}(OXYGEN-[O2])$	E, M
8	O2 →	$R_{res}=k_{res}[FS]\frac{\left[O2\right]}{K_{res(O2)}+[O2]}$	M
9	F2 → F3	$R_{23}=k_{23}\frac{\left[F2\right]}{K_{23(F2)}+[F2]}[OXYGEN]$	V

The model describes a population of yeast cells growing exponentially on two nutrients called IRON and OXYGEN. The exponential growth rate of WT cells, given by parameter *α*_*cell*_, was set at $0.00\bar{3}$ min^-1^ which corresponds to a typical growth rate of respiring WT yeast cells [21]. The *in-silico* cell was subdivided into 3 compartments, including cytosol (Fig 1, blue), mitochondria (yellow), and vacuoles (green); respective fractional volumes were *f*_*cyt*_ = 0.8, *f*_*mit*_ = 0.1 and *f*_*vac*_ = 0.1. IRON enters the cell at rate ***R***_***cyt***_ where it becomes cytosolic iron **FC**. (Model components and rates are introduced in bold.) OXYGEN enters the cell at rate ***R***_***O2***_ where it becomes mitochondrial **O2**. FC enters mitochondria at rate ***R***_***mit***_, becoming a labile pool of Fe^II^ called **FM**. FC can also enter vacuoles at rate ***R***_***vac***_, becoming a pool of Fe^II^ called **F2**. FC can also metallate all ISC proteins in the cytosol and nucleus, forming component **CIA** in accordance with rate ***R***_***cia***_. In this model, CIA reflects all of the iron from the labile pool that is delivered to cellular sites other than mitochondria or vacuoles. In the mitochondria, FM is the substrate for generating hemes and ISCs (collectively called **FS**), at rate ***R***_***isu***_. This reaction is inhibited by O2 as evidenced by the additional term in the rate-law expression. In that expression [O2]_sp_ represents the set-point concentration for O2 inhibition. FS is a catalyst for respiration (involving substrate O2 which reacts at rate ***R***_***res***_); this limits O2 from diffusing into the mitochondria where it reacts with FM to generate nanoparticles (**MP**) at rate ***R***_***mp***_. In the vacuole, F2 can be oxidized to Fe^III^, called **F3**, in accordance with rate ***R***_***23***_. The resulting ODEs in (1) are given in terms of reaction rates.


$$\left\{\begin{array}{l} {}[FC]'=R_{cyt}-R_{vac}-R_{mit}-R_{cia}-D_{FC}\\ {}[CIA]'=R_{cia}-D_{CIA}\\ {}[F2]'=\frac{f_{cyt}}{f_{vac}}R_{vac}-R_{23}-D_{F2}\\ {}[F3]'=R_{23}-D_{F3}\\ {}[FM]'=\frac{f_{cyt}}{f_{mit}}R_{mit}-R_{isu}-R_{mp}-D_{FM}\\ {}[FS]'=R_{isu}-D_{FS}\\ {}[MP]'=R_{mp}-D_{MP}\\ {}[O2]'=R_{O2}-R_{mp}-R_{res}-D_{O2} \end{array}\right\}$$ (1)

The last term in each ODE reflects the dilution of the designated component due to cell growth. Fractional volumes augment rates of interregional reactions in which reactants and/or products are in different cellular regions. *R*_*vac*_ and *R*_*mit*_ refer to rates *in the cytosol*. To calculate corresponding rates for the same interregional reactions in vacuoles and mitochondria, cytosolic rates must be multiplied by the corresponding fractional volume ratios, as in (1). If this were not done, mass would not be conserved.

Our first objective was to determine these rates. After including the fractional volume ratios given above, the ODEs in (1) were organized into matrix form yielding (2)

$$\left[\begin{array}{ccccccccccccccccc} 1 & -1 & -1 & -1 & 0 & 0 & 0 & 0 & 0 & -1 & 0 & 0 & 0 & 0 & 0 & 0 & 0\\ 0 & 0 & 0 & 1 & 0 & 0 & 0 & 0 & 0 & 0 & -1 & 0 & 0 & 0 & 0 & 0 & 0\\ 0 & 0 & 8 & 0 & 0 & 0 & 0 & -1 & 0 & 0 & 0 & -1 & 0 & 0 & 0 & 0 & 0\\ 0 & 0 & 0 & 0 & 0 & 0 & 0 & 1 & 0 & 0 & 0 & 0 & -1 & 0 & 0 & 0 & 0\\ 0 & 8 & 0 & 0 & -1 & -1 & 0 & 0 & 0 & 0 & 0 & 0 & 0 & -1 & 0 & 0 & 0\\ 0 & 0 & 0 & 0 & 1 & 0 & 0 & 0 & 0 & 0 & 0 & 0 & 0 & 0 & -1 & 0 & 0\\ 0 & 0 & 0 & 0 & 0 & 1 & 0 & 0 & 0 & 0 & 0 & 0 & 0 & 0 & 0 & -1 & 0\\ 0 & 0 & 0 & 0 & 0 & -1 & 1 & 0 & -1 & 0 & 0 & 0 & 0 & 0 & 0 & 0 & -1 \end{array}][\begin{array}{c} R_{cyt}\\ R_{mit}\\ R_{vac}\\ R_{cia}\\ R_{isu}\\ R_{mp}\\ R_{O2}\\ R_{23}\\ R_{res}\\ D_{FC}\\ D_{CIA}\\ D_{F2}\\ D_{F3}\\ D_{FM}\\ D_{FS}\\ D_{MP}\\ D_{O2} \end{array}]=[\begin{array}{c} FC'\\ CIA'\\ F2'\\ F3'\\ FM'\\ FS'\\ MP'\\ O2' \end{array}\right]$$ (2)

where the 8×17 stoichiometric (or ***S***) matrix is multiplied by a vector of rates (the ***R*** vector) to yield a vector of component concentration derivatives. To separate rates into independent and dependent groups, the ***S*** matrix was transformed into the Reduced-Row-Echelon Form (RREF), as shown on the left-hand-side of (3).

$$[\begin{array}{ccccccccccccccccc} 1 & 0 & 0 & 0 & 0 & 0 & 0 & 0 & 0 & -1 & -1 & -\frac{1}{8} & -\frac{1}{8} & -\frac{1}{8} & -\frac{1}{8} & -\frac{1}{8} & 0\\ 0 & 1 & 0 & 0 & 0 & 0 & 0 & 0 & 0 & 0 & 0 & 0 & 0 & -\frac{1}{8} & -\frac{1}{8} & -\frac{1}{8} & 0\\ 0 & 0 & 1 & 0 & 0 & 0 & 0 & 0 & 0 & 0 & 0 & -\frac{1}{8} & -\frac{1}{8} & 0 & 0 & 0 & 0\\ 0 & 0 & 0 & 1 & 0 & 0 & 0 & 0 & 0 & 0 & -1 & 0 & 0 & 0 & 0 & 0 & 0\\ 0 & 0 & 0 & 0 & 1 & 0 & 0 & 0 & 0 & 0 & 0 & 0 & 0 & 0 & -1 & 0 & 0\\ 0 & 0 & 0 & 0 & 0 & 1 & 0 & 0 & 0 & 0 & 0 & 0 & 0 & 0 & 0 & -1 & 0\\ 0 & 0 & 0 & 0 & 0 & 0 & 1 & 0 & -1 & 0 & 0 & 0 & 0 & 0 & 0 & -1 & -1\\ 0 & 0 & 0 & 0 & 0 & 0 & 0 & 1 & 0 & 0 & 0 & 0 & -1 & 0 & 0 & 0 & 0 \end{array}][\begin{array}{c} R_{cyt}\\ R_{mit}\\ R_{vac}\\ R_{cia}\\ R_{isu}\\ R_{mp}\\ R_{O2}\\ R_{23}\\ R_{res}\\ D_{FC}\\ D_{CIA}\\ D_{F2}\\ D_{F3}\\ D_{FM}\\ D_{FS}\\ D_{MP}\\ D_{O2} \end{array}]=[\begin{array}{c} 0\\ 0\\ 0\\ 0\\ 0\\ 0\\ 0\\ 0 \end{array}].$$ (3)

Starting from the top left side, the RREF matrix has a staircase pattern of 1’s and 0’s with a “pivot” at each step. There are 8 pivot columns which correspond to *dependent* rates; these include *R*_*cyt*_, *R*_*mit*_, *R*_*vac*_, *R*_*cia*_, *R*_*isu*_, *R*_*mp*_, *R*_*o2*_, and *R*_*23*_. The 9 remaining non-pivot columns correspond to *independent* rates; these include *R*_*res*_, *D*_*FC*_, *D*_*CIA*_, *D*_*F2*_, *D*_*F3*_, *D*_*FM*_, *D*_*FS*_, *D*_*MP*_, and *D*_*O2*_. Independent rates can be assigned any value. However, once assigned, dependent rates can be determined directly from the 8 relationships obtained from the null space of the RREF, Eq (4), one for each component.


$$\left\{\begin{array}{l} R_{cyt}=D_{FC}+D_{CIA}+\frac{1}{8}D_{F2}+\frac{1}{8}D_{F3}+\frac{1}{8}D_{FM}+\frac{1}{8}D_{FS}+\frac{1}{8}D_{MP}\\ R_{mit}=\frac{1}{8}D_{FM}+\frac{1}{8}D_{FS}+\frac{1}{8}D_{MP}\\ R_{vac}=\frac{1}{8}D_{F2}+\frac{1}{8}D_{F3}\\ R_{cia}=D_{CIA}\\ R_{isu}=D_{FS}\\ R_{mp}=D_{MP}\\ R_{o2}=R_{res}+D_{MP}+D_{O2}\\ R_{23}=D_{F3} \end{array}\right\}$$ (4)

The null space includes subsets of reaction rate vector ***R*** for which the system is at steady-state such that component concentrations are unchanging (i.e. [C_i_]’ = 0). Unlike simple systems which possess a single steady-state, more complex systems may have many such states. A steady-state condition is guaranteed when all null space relationships are obeyed. However, this does not guarantee that the steady-state will be stable or employed by real cells.

The dimension of the null space is given by the number of independent rates, 9 in this case. The lower the dimension of this space, the fewer steady-state solutions that exist; if the null space were zero-dimensional, there would be a unique steady-state solution given by the zero vector. In our case, 8 of those dimensions represent rates of dilution. Since the exponential growth rate of cells (called *α*_*cell*_) has been measured experimentally, dilution rates *could* be assigned *if* the steady-state concentrations of each component were known. This would lower the dimensionality of the null space to 1 in which case only *R*_*res*_ would need to be freely chosen to obtain a unique steady-state solution.

### Defining cellular states

From a biological perspective, a cellular state is defined by a particular genetic strain (*internal* conditions) grown in a specified nutrient environment (*external* conditions). Here, an internal cellular state (W, Y or D) was defined by a set of rate-constants, Michaelis-Menten parameters, and the exponential growth rate of the cells. External conditions included fixed [IRON] and [OXYGEN] concentrations. For non-WT strains or conditions, a user-defined change in an internal parameter was taken to be the *primary mutation*, and all other resulting internal changes were viewed as *responses* to the primary change. The primary mutation in the Y state involved reducing *k*_*isu(W)*_ and *α*_*cell(W)*_ by factors of 10× and 2×, respectively, relative to in the W state. *k*_*isu*_ was primarily affected by the absence of Yfh1 because this protein is part of a catalyst driving the reaction FM → FS. Experimentally, *α*_*cell*_ declines under Y conditions, from *α*_*cell(w)*_ = $0.00\bar{3}$ min^-1^ when *k*_*isu(W)*_ = $6.\bar{6}$ min^-1^, to *α*_*cell(Y)*_ = $0.001\bar{6}$ min^-1^ when *k*_*isu(Y)*_ = $0.\bar{6}$ min^-1^. These were the only two primary changes needed to cause the shift W → Y. As the cell transitioned, its growth rate was related to the changing value of *k*_*isu(W→Y)*_ according to relationship (5).


$$\alpha_{cell(W\to Y)}=(\frac{\alpha_{cell(W)}-\alpha_{cell(Y)}}{k_{isu(W)}-k_{isu(Y}{}_{)}})k_{isu(W\to Y)}+(\frac{\alpha_{cell(Y)}k_{isu(W)}-\alpha_{cell(W)}k_{isu(Y}{}_{)}}{k_{isu(W)}-k_{isu(Y}{}_{)}})$$ (5)

The D state was not generated by a primary mutation; rather the nutrient IRON concentration was incrementally reduced from 40 → 1 μM as the cell transitioned from W → D.

For the current study, the phenotype associated with a cell state referred to differences in steady-state component concentrations relative to in W cells; this was the only “observable”. More sophisticated phenotypes may eventually be defined by differences in stabilities, sensitivities to perturbations, cellular morphology, growth rates, etc.

### Steady-state concentrations

The steady-state concentrations assumed for the three cellular states are given in Table 2. Concentrations were estimated from experimental studies primarily using Mössbauer spectroscopy and iron-concentration determinations of whole cells, isolated mitochondria, isolated vacuoles, and isolated cytosol for the three cellular states; see Appendix C in S1 Text.

**Table 2:** Experimental (estimates) and calculated steady-state concentrations without logistic functions for each cellular state. Concentrations for individual components are local and are given in units of μM. Data were estimated as described in Appendix C in S1 Text Simulated concentrations were within 1% of experimental values. The [Fe_cell_] values listed are obtained by summing the concentrations of individual iron-containing component multiplied by the fractional volume (0.8 for cyt; 0.1 for vac; 0.1 for mit), [Fe_cell_] = *f*_*cyt*_[CIA] + *f*_*vac*_[F2] + *f*_*vac*_[F3] + *f*_*cyt*_[FC] + *f*_*mit*_[FM] + *f*_*mit*_[FS] + *f*_*mit*_[MP]. For the first column, [Fe_cell_] = 0.8⋅80 + 0.1⋅200 + 0.1⋅3400 + 0.8⋅20 + 0.1⋅100 + 0.1⋅500 + 0.1⋅50 = 505 μM. Comparable computed values as obtained by Fernandez et al. [20] are given on the right side of the table for comparison. Predictions for the H_W_ and H_Y_ states assumed CRM case 1 autoregulation. H_w_ and H_Y_ state values were not used to train the model.

State (columns) → Component (rows)↓ (μM)	W	Y	D	H_W_	H_Y_	Fernandez (W)	Fernandez (Y)	Fernandez (D)	Fernandez (H_W_)	Fernandez (H_Y_)
CIA	80	70	68	174	100	42	62.5	42.3	6.6	37.5
F2	200	30	20	1736	5500	300	32.6	20.5	6500	4820
F3	3400	420	60	1194	100	5900	0.03	435	404	11.1
FC	20	10	5	16.8	18.5	7	328	2.76	6.6	2075
FM	100	200	50	44.7	467	290	482	160	277	4560
FS	500	150	300	545	273	597	0.09	392	639	25.9
MP	50	8500	20	1.93	1129	42	43,270	40.5	1.95	634
O2	1.0	0.48	1.2	0.13	0.0051	0.17	59.8	0.25	0.01	0.15
α_cell_ (min^-1^)	0.003333	0.0020	0.003333	0.003333	0.0020	0.003333	0.002	0.003	0.00333	0.00333
[Fe_cell_]	505	994	103.4	505	842	940	4700	141	880	2700
[IRON]	40	40	1.000	40	40	41	41	1	41	41
[OXYGEN]	100	100	100	25	1	~100	~100	~100	~25	~25

Although we did not formally evaluate identifiability for this model, we constructed the model mindful of this principle. We kept it simple, and made sure that each iron-containing component was directly connected to experimental measurements. The only component that has not been measured experimentally is O2 (dissolved O_2_ concentration in the mitochondrial matrix). However, there is substantial indirect evidence that this space is hypoxic in healthy mitochondria (argued in [37]).

We integrated all such information to generate the listed concentrations for each component and for whole cells. This constrained the system significantly. Nevertheless, these concentrations should be viewed as best-approximations or informed hypotheses due to their large uncertainties. Components with high concentrations (e.g. MP in the Y state or F3 in the W state) were known with greater accuracy than those with low concentrations (e.g. FC).

Using those assigned steady-state concentrations, dilution rates of each component were calculated by multiplying each steady-state concentration by *α*_*cell*_. The assumed value of the remaining independent rate (*R*_*res*_) was estimated from the literature. We initially selected *R*_*res*_ = 10,000 μM/min based on the results of Popel et al [38]; see Appendix A in S1 Text for detailed considerations. Dependent rates were then calculated by solving (4). Resulting reaction rates are listed in Table 3.

**Table 3:** Steady-state rates, rate-constants, and *K*_*m*_ parameters for each defined state. Rates are given in units of μM/min. *K*_*m*_ values are in units of μM. Units for rate-constants are dictated by the associated rate-law expressions (Table 1). Excessive digits are given to allow accurate simulations. Far fewer digits are significant biologically. The same situation applies for the numbers given in all tables.

Parameter	W	Y	D	H_W_	H_Y_
*R*_*cia*_	0.266640	0.140000	0.226644	0.581018	0.200896
*R*_*23*_	11.332200	0.840000	0.199980	3.980788	0.198875
*R*_*isu*_	1.666500	0.300000	0.999900	1.817023	0.546340
*R*_*mp*_	0.166650	17.000000	0.066660	0.006415	2.203349
*R*_*vac*_	1.499850	0.112500	0.033330	1.220877	1.405497
*R*_*mit*_	0.270806	2.212500	0.154151	0.246539	0.460551
*R*_*cyt*_	2.103960	2.485000	0.430790	2.104468	2.104030
*R*_*res*_	9090.909091	1772.360000	6000.000000	2322.757108	49.979927
*R*_*o2*_	9091.080000	1789.360000	6000.070000	2322.770833	52.183199
*D*_*FC*_	0.066660	0.020000	0.016665	0.056035	0.037089
*D*_*CIA*_	0.266640	0.140000	0.226644	0.581002	0.200634
*D*_*F2*_	0.666600	0.060000	0.066660	5.786121	10.999540
*D*_*F3*_	11.332200	0.840000	0.199980	3.981002	0.199939
*D*_*FM*_	0.333300	0.400000	0.166650	0.148875	0.934662
*D*_*FS*_	1.666500	0.300000	0.999900	1.817025	0.546342
*D*_*MP*_	0.166650	17.000000	0.066660	0.006422	2.258360
*D*_*O2*_	0.003333	0.000963	0.004074	0.000442	0.000010
*k*_*cia*_	0.533280	0.420000	1.133219	1.272209	0.417560
*k*_*23*_	0.226643	0.064400	0.021998	0.177576	0.206108
*k*_*isu*_	6.666000	0.666600	6.666000	6.666000	0.666600
*k*_*mp*_	0.001667	0.176597	0.001091	0.001082	0.932340
*k*_*vac*_	2.999702	0.337501	0.166650	2.673257	2.921314
*k*_*mit*_	0.541613	6.637541	0.770759	0.539827	0.957252
*k*_*cyt*_	2.629947	3.106247	4.738685	2.630585	2.630037
*k*_*res*_	36.363636	36.363636	36.363636	36.363636	36.363636
*k*_*O2*_	91.829091	17.980069	60.743001	93.406707	52.448426
*K*_*cyt(IRON)*_	10	Same	Same	Same	Same
*K*_*mit(FC)*_	20	Same	Same	Same	Same
*K*_*vac(FC)*_	20	Same	Same	Same	Same
*K*_*cia(FC)*_	20	Same	Same	Same	Same
*K*_*23(F2)*_	200	Same	Same	Same	Same
*K*_*isu(FM)*_	100	Same	Same	Same	Same
*K*_*res(O2)*_	1.0	Same	Same	Same	Same
[O2]_sp_	1.0	Same	Same	Same	Same

### Kinetic constants

Once a complete set of reaction rates was generated, each rate was equated to its corresponding rate-law expression as given in Table 1. The rate-constant associated with each rate was calculated using the steady-state concentrations in Table 2. The Michaelis-Menten *K*_*m*_ parameter for each rate-law expression was initially equated to the concentration of the corresponding substrate for the W state: i.e. *K*_*m(w)*_ = *K*_*m(Y)*_ = *K*_*m(D)*_ = [C_i_]_(w)_. This assumption rendered the rate of the designated reaction most sensitive to changes in [C_i_]. It also reduced the number of unknown parameters and allowed the corresponding rate-constants (generically called *k*_*obs*_) to be solved. The *K*_*m*_ parameter *K*_*cyt(IRON)*_ was minorly adjusted from its initial assumed value to avoid instability. Rate-constant values are organized in Table 3. The 3 sets of rate-constants, one for each cellular state, and the corresponding sets of steady-state concentrations, one for each component, along with α_cell_ values, defined the W, Y and D states.

### Stability of the System

A fundamental characteristic of biochemical processes occurring within actual growing cells is their *stability to modest or limited perturbations*. Any component in the cell whose concentration at some time differs from its steady-state concentration will eventually return to that concentration. There are limits to the ability of the system to recover, such that a perturbation of sufficient magnitude can cause the system to attract to a different steady-state (or to an unstable state). Correspondingly, in real cells, extreme perturbations can be lethal. To assess whether the calculated steady-states in our *in-silico* cell were stable, each state was evaluated by constructing the Jacobian (***J***) matrix and solving its eigenvalues. Such states are stable to perturbations if and only if all eigenvalues of the ***J*** matrix lie in the left half of the complex plane. To construct ***J***, the rates given in the ODEs of (1) were replaced with the rate-law expressions in Table 1, generating the functions in Table 4. The partial derivatives of those functions with respect to each component of the system were obtained and organized into matrix form. Values for [C_i_], *k*_*obs*_, and *K*_*m*_ parameters were included in calculating matrix elements. Since the model included 8 ODEs and 8 components, the ***J*** matrix was 8 × 8. Matrix elements equaled 0 when a component was not included in the ODE. Eigenvalues were then calculated by solving (6)

det|J−λI|=0 (6)

where *I* is the identity matrix and *λ* is a vector of eigenvalues. For each cellular state, the complete set of eigenvalues are required to be negative for the system to be stable to perturbation in component concentrations. Using *R*_*res*_ = 10,000 μM/min, not all eigenvalues were negative. This illustrates that although any value of *R*_*res*_ can be selected to satisfy the null space relationships, some values may not result in a system that is stable from perturbations. In this case, *R*_*res*_ = 9090 μM/min was selected since it was near to our initial selection yet afforded a system in which all eigenvalues were negative (Table 5).

**Table 4:** Functions used in constructing the Jacobian matrix.

name	Function
$\frac{d[FC]}{dt}=f_{ODEFC}=$	$\frac{k_{cyt}[IRON]}{K_{Cyt(IRON)}^{}+[IRON]}-\frac{k_{vac}[FC]}{K_{vac(FC)}^{}+[FC]}-\frac{k_{mit}[FC]}{K_{mit(FC)}+[FC]}-\frac{k_{cia}[FC]}{K_{cia(FC)}^{}+[FC]}-\alpha_{cell}[FC]$
$\frac{d[CIA]}{dt}=f_{ODECIA}=$	$\frac{k_{cia}[FC]}{K_{cia(FC)}^{}+[FC]}-\alpha_{cell}[CIA]$
$\frac{d[F2]}{dt}=f_{ODEF2}=$	$\frac{f_{cyt}}{f_{vac}}\frac{k_{vac}[FC]}{K_{vac(FC)}^{}+[FC]}-k_{23}\frac{\left[F2\right]}{K_{23(F2)}+[F2]}[OXYGEN]-\alpha_{cell}[F2]$
$\frac{d[F3]}{dt}=f_{ODEF3}=$	$k_{23}\frac{\left[F2\right]}{K_{23(F2)}+[F2]}[OXYGEN]-\alpha_{cell}[F3]$
$\frac{d[FM]}{dt}=f_{ODEFM}=$	$\frac{f_{cyt}}{f_{mit}}\frac{k_{mit}[FC]}{K_{mit(FC)}^{}+[FC]}-\frac{\left(k_{isu}[FM]\right)}{K_{isu(FM)}+[FM]}-k_{mp}[FM][O2]-\alpha_{cell}[FM]$
$\frac{d[FS]}{dt}=f_{ODEFS}=$	$\frac{k_{isu}[FM]}{K_{isu(FM)}^{}+[FM]}(\frac{{\left[O2\right]}_{sp}}{{\left[O2\right]}_{sp}+[O2]})-\alpha_{cell}[FS]$
$\frac{d[MP]}{dt}=f_{ODEMP}=$	$k_{mp}[FM][O2]-\alpha_{cell}[MP]$
$\frac{d[O2]}{dt}=f_{ODEO2}=$	$k_{O2}(OXYGEN-[O2])-k_{mp}[FM][O2]-k_{res}[FS]\frac{\left[O2\right]}{K_{res(O2)}^{}+[O2]}-\alpha_{cell}[O2]$

**Table 5:** Eigenvalues λ_i_ of the Jacobian matrices. Calculation were from Wolfram using CRM case 1. Units are min^-1^.

State	λ_FC_	λ_CIA_	λ_F2_	λ_F3_	λ_FM_	λ_FS_	λ_MP_	λ_O2_
CRM not included								
W	-4637.46	-0.0542654	-0.0316635	-0.011413	-0.003333	-0.003333	-0.003333	-0.0019854
Y	-2539.03	-0.166333	-0.0872849	-0.0263478	-0.002	-0.002	-0.002	-0.0000970953
D	-2269.9	-0.069593	-0.0150332	-0.012423	-0.003333	-0.003333	-0.003333	-0.00233368
								
CRM included								
W	-4637.46	-0.072625	-0.0316634	-0.0133618	-0.003333	-0.003333	-0.003333	-0.0022985
Y	-2539.04	-0.880372	-0.501518	-0.0263477	-0.0028465	-0.002	-0.002	-0.002
D	-2269.89	-0.08787	-0.012423	-0.00514911 +0.00639993*i*	-0.00514911 -0.00639993*i*	-0.003333	-0.003333	-0.003333

### Solving the dynamical system

With all kinetic parameters and concentrations assigned, and stability toward perturbations confirmed, the ODE systems were integrated to exhibit time-dependent behavior. Steady-states were assumed to be the dynamical component concentrations attained at 50,000 min. Computations were performed in Wolfram Mathematica notebooks along with Python, and JavaScript (to determine nonlinear regression values). States were modeled with the same ODE system but with parameters and variables assigned to their state-specific values (Tables 2 and 3). Steady-state concentrations, as obtained by simulations, matched the experimental values in Table 2 to within 1% accuracy.

We defined a cellular steady-state not merely as being in the null space of the stoichiometric matrix but also being stable to perturbations and affording the set of steady-state concentrations for that state (W, Y, or D) given in Table 4.

### Including autoregulation

The next challenge was to model transitions from the W state to either the Y or D state. This was equivalent to having a growing healthy cell abruptly develop a mutation in the *YFH1* gene, causing it to become diseased, or for an iron-replete cell to be abruptly placed in iron-deficient media. Rather than manually changing the set of rate constants from those that defined the W state to those that defined Y or D and then allow the system to evolve in time, we wanted to design a cellular regulatory mechanism (CRM) which would facilitate such transitions automatically in response to the primary or causal event(s). In our cases, this meant either altering *k*_*isu*_ and α_cell_ for the W → Y transition or altering [IRON] for the W → D transition. At that point, the system should respond in time to ultimately generate the Y or D state.

Most reactions in a cell are enzyme-catalyzed and regulated at the transcriptional level. However, the enzymes catalyzing the coarse-grain reactions of Fig 1 were not explicit components of the model. Consequently, the level of gene expression for these assumed enzymes was viewed as being reflected in the rate-constants associated with the considered reactions. Regulating rate-constants more accurately simulates changes in gene expression levels of an enzyme than would regulating rates *per se*, as the latter would be affected by substrate concentrations according to their corresponding rate-law expressions, whereas the former would not.

Tyson et al. suggested using “soft” (a.k.a. continuous) Heaviside logistical functions to regulate reactions within an ODE framework [3]; this would allow the sensitivity of the regulatory response and the strength of the regulatory interaction to be easily adjusted. This framework was included in our model by dividing observed rate-constants for each cellular state W, Y, and D into two components; a regulated (or inducible) term (*k*_*reg*_) and an unregulated (or constitutive) term (*k*_*unreg*_), as in (7).


$$k_{obs}=k_{reg}\frac{1}{1+e^{n([SP]-[Sen])}}+k_{unreg}$$ (7)

Here, *Sen* is the sensed species (or sensor), *SP* is the “setpoint” concentration of *Sen*, and *n* controls the sensitivity of the response. When [Sen] = [SP], expression for the regulated gene would be half-maximal. Including these logistical functions rendered *k*_*obs*_ a function of time because [Sen] is a function of time. If *k*_*obs*_ for a given reaction differed in each cellular state, that difference was attributed to changes in the gene expression level of the implicit enzyme catalyzing the reaction.

For the system to be *auto*regulated, *Sen* must be a component of the model (i.e. FC, FM, FS, MP, O2, F2, F3, or CIA), *not* a user-controlled parameter external to the model. In this way, the changing concentration of *Sen* would automatically regulate the system regardless of cellular state. For each regulated reaction, *Sen* had to be identified and values for *k*_*reg*_, *k*_*unreg*_, *n*, and *SP* assigned.

To do this, we first assumed that differences in *k*_*obs*_ from one cellular state to another arose entirely from the regulated part of (7), specifically due to changes in [Sen] with *k*_*reg*_, *k*_*unreg*_, [SP] and *n* fixed for the same reaction in all three cellular states. Other regulated reactions could employ the same or different *Sen* without restriction. Also, if the same *Sen* were used, values for *k*_*reg*_, *k*_*unreg*_, [SP], and *n* might differ. Identifying which component would best regulate the system as it attempted to transition from one state to another–i.e. finding the best *Sen*—became a major challenge.

Both time-dependent and steady-state (time-independent) cell-state transitions were considered. Time-dependent transitions were generated by changing, at some moment in time, internal and/or external parameters from those characterizing the healthy W state to those characterizing the diseased Y state or iron-deficient D state. Time-independent steady-state transitions from W → Y or W → D were generated by incrementally (linearly) changing two parameters (*k*_*isu*_ and *α*_*cell*_) for the W → Y transition, and one parameter [IRON] for the W → D transition. In both steady-state cases, at each increment along the transition, the system was allowed to evolve 50,000 min ensuring that this condition was established.

### Evolution of cellular regulatory mechanisms

The next challenge was to identify the “best” regulatory mechanism to apply to each regulatable reaction in the model, and to justify or define what is meant by “best”. We employed a process, analogous to Darwinian evolution, in which all possible cellular regulatory mechanisms (CRMs) were considered, and then subsets of those cases were selected based on their ability to satisfy six sequentially applied fitness criteria (filtering).

**i) *Uniqueness*:** In principle each of the 9 rate-constants in the model could be regulated by any of the 8 model components. This afforded 8^9^ = 134,217,728 possible CRMs. The first filter, called *uniqueness*, stipulated that the magnitude of *k*_*obs*_ for a given reaction should be unique to a given cellular state; if not, the reaction need not be regulated. Thus, only reactions for which *k*_*obs(W)*,_ *k*_*obs(Y)*_ and *k*_*obs(D)*_ had distinct values were *regulatable*. According to Table 3, this included 7 of the 9 reactions (all except *R*_*isu*_ and *R*_*res*_). *k*_*isu*_ was not autoregulated in this model as it was the primary mutation for generating the Y state and was manually assigned the same value for both W and D states. *k*_*res*_ was constant for all three states. After applying this filter, 8^7^ = 2,097,152 CRM cases were selected for further screening.

**ii) *Trending*:** The concentration of *Sen* must either trend *with* changes in rate-constants (for feedforward regulation) or trend *against* them (for feedback regulation). For a given reaction with rate-constant *k*_*obs*_, this condition is described by the rule

$$\begin{array}{l} Trend\;in\;k_{obs}:\;k_{obs(i)}>k_{obs(j)}>k_{obs(k)}\\ Feedback\;Trend:\;[Sen_{\left(i\right)}]<[Sen_{\left(j\right)}]<[Sen_{\left(k\right)}]\\ Feedforward\;Trend:\;[Sen_{\left(i\right)}]>[Sen_{\left(j\right)}]>[Sen_{\left(k\right)}] \end{array}$$ (8)

where *i*, *j*, and *k* refer to W, Y, and D states (no order implied). A single deviation from either trending rule (feedback or feedforward) disqualified the CRM entirely. Of 2,097,152 CRMs screened, only 576 survived.

**Fig 2:**
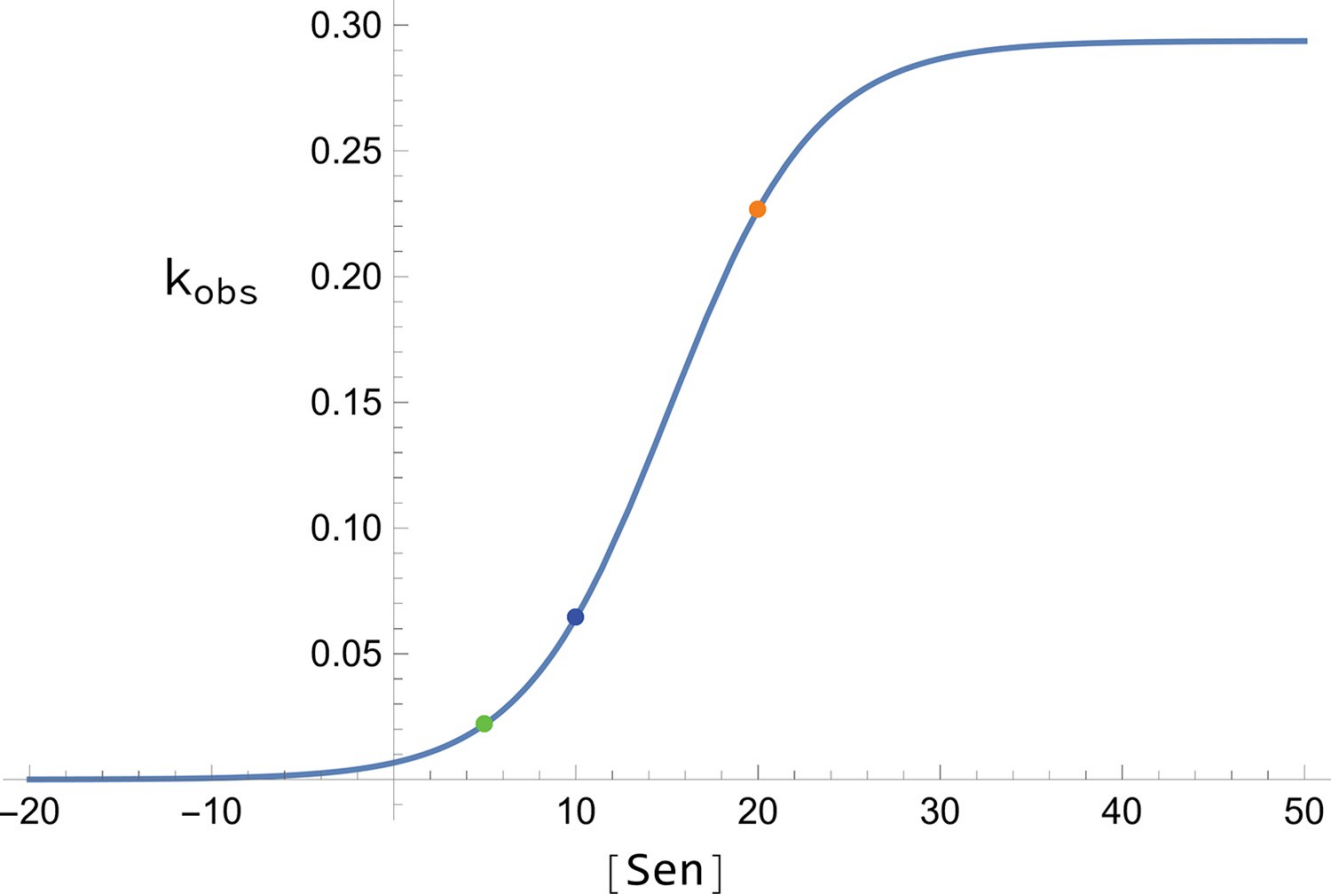
Behavior of continuous Heaviside Logistical functions in regulating rate-constants. In this particular plot the ordinate is *k*_*23*_ (as an example of a regulated *k*_*obs*_) while the abscissa is [FC] (example of [Sen]). The solid blue line is the function calculated by nonlinear regression using parameters in Table 6. Orange, blue and green dots indicate *k*_*23*_ for W, Y, and D states, respectively, as given in Table 3.

**iii) *Targeting*:** At this point, *Sen* could be assigned for a given logistic function, but the behavior of that function would change depending on how [Sen] changed (either [Sen]_ss_ or [Sen]_t_). The behavior would also change depending on the values of *k*_*reg*_, *k*_*unreg*_, *n*, and [SP]. Best-fit values for these parameters (Table 3) were obtained using nonlinear regression, minimizing the difference between *k*_*obs*_ and the respective rate-constant values for a state. Essentially, a logistic function mapped a state-specific component concentration to a state’s rate-constant value as illustrated in Fig 2. Nonlinear regression optimization was done via Desmos’s algorithm as described at https://engineering.desmos.com/articles/regressions-improvements/.

The targeting error for a given component was the difference between the simulated steady-state concentration for the Y or D state, and the experimentally estimated steady-state concentrations given in Table 2, normalized to the experimental concentrations.


$$\mathrm{Target}\_\mathrm{Err}={\left|\frac{{\left[C_{i}\right]}_{\left(Y,Dsim\right)}-{\left[C_{i}\right]}_{\left(Y,D\exp\right)}}{{\left[C_{i}\right]}_{\left(Y,D\exp\right)}}\right|}_{t=50,000}\leq 0.01$$ (9)

The maximum allowed Target_Err for any component and any transition was set at ≤ 1%. The Desmos (desmos.com) algorithm was used for nonlinear regression such that no initial values were required. Desmos performs regression problems using the Levenberg-Marquardt algorithm which minimizes the sum-of-squares and converges to a minimum (local or global) solution. [SP] values < 0 or > 10,000 were excluded as chemically impossible or unreasonable, respectively. The resulting pool of 18 Heaviside functions could be combined as needed to construct a given CRM. Only final steady-state concentrations for a component were included in solving a particular Heaviside function; this allowed us to “mix and match” such functions for testing the validity of any candidate CRM. The case was excluded if Target_Err was > 1% for any component. Of the 576 CRMs examined, 146 survived. An example of a CRM excluded due to poor *Targeting* is given in Fig A in S1 Text.

**iv) *Wandering*:** Depending on the CRM, a component concentration plot might deviate from a straight-line transition from the steady-state concentration in the W state to the steady-state concentration in Y or D states. W → Y or W → D transitions that minimized “wandering” were preferred. This preference was based on *Occam’s razor*. To quantify the extent of wandering, we calculated the arc length of the concentration transition lines in steady-state plots as described [39]. Component *C*_*i*_ in time-dependent transitions generally approached an asymptotic limit by 5000 min, and thus the arc length for each component was determined over this period using (10).


ALt(Ci)=∫050001+[Ci'(t)]2dt (10)

To implement this equation, steady-state transitions were divided into 100 increments j = 1…100, assuming a linear incremental change in parameter *k*_*isu*_ and α_cell_ for W→Y, and in parameter [IRON] for W→D. For each *C*_*i*_, arc length was defined as the sum of the Euclidean distances to the next point.


$${\mathrm{AL}}_{\mathrm{ss}}(C_{i,ss})=\sum_{j=1}^{j=99}\sqrt{{\left(C_{i,j+1}-C_{i,j}\right)}^{2}+{\left(k_{isu,j+1}-k_{isu,j}\right)}^{2}}$$ (11)

For a given component, the associated percentage error for steady-state transitions was

$${\mathrm{Wander}}_{\mathrm{ss}}\_\mathrm{Err}(C_{i,ss})=\frac{AL{\left(C_{i}{}_{,ss}\right)}_{sim}-AL{\left(C_{i}{}_{,ss}\right)}_{\min}}{AL{\left(C_{i}{}_{,ss}\right)}_{\min}}\times 100$$ (12)

For each component of a given CRM, Wander_Err for steady-state and time transitions were averaged separately, and the lowest-error half of the 146 cases were selected for each transition type. Each set contained approximately 73 cases. The intersection of those sets contained 26 cases, and these were selected. An example of a CRM excluded due to excessive *Wandering* is given in Fig B in S1 Text. This filter (and the Smoothness filter) favor cases in which oscillations are minimized.

**v) *Smoothness*:** This filter was designed to exclude CRMs in which steady-state transition plots contained abrupt nonphysical spikes. Smoothness was essentially the normalized arc-length for any point in a plot. A smoothness error was calculated as the defining model parameter was changed (*k*_*isu*_ and *α*_*cell*_ for W→Y or [IRON] for W→D) linearly over 100 increments j = 1…100. The smoothness error for a given CRM was determined by summing all “spikes” (normalized distance or arc-length between two points) for each component concentration in the steady-state transition from W to either Y or D, as determined by (13).


$$\mathrm{Sm}\_\mathrm{Err}=\sum_{i=1}^{8}100\sum_{j=1}^{99}\frac{\left|C_{i}(k_{isu}{}_{,j})-C_{i}(k_{isu,j+1})\right|}{C_{i}{\left(k_{isu}{,}_{j}\right)}_{i}}$$ (13)

Sm_Err for an individual CRM was defined to be the sum of the W → D and W → Y smoothness error scores. We wanted to select CRMs that allowed both transitions to occur smoothly. Of the 26 cases considered, the top 2 cases had similar smoothness scores, and so both were selected. In situations involving many cases, clustering may be required to identify the best group. The process of clustering would start by plotting the smoothness scores on a number line and see which cases grouped together. Clusters are separated by large gaps in the number line where no values reside. In our case, we applied mean-shift clustering (calculated using Python’s scikit-learn library) on our 26 cases and arrived at a similar selection. An example of a CRM which was excluded due to insufficient *Smoothness* is given in Fig C in S1 Text.

**vi) *n/SP-Reasonableness*:** Parameter *n* in (7) represents the sensitivity of the regulation or the steepness of the Heaviside function plotted vs ([SP]–[Sen]). In real systems, sensitivities reflect cooperative binding of transcription factor proteins onto DNA promotor sites. Following Occam’s Razor, values of *n* nearer to 1 were preferred, as this avoided the implication of cooperativity. We calculated the error associated with *n* deviating from 1 as

n_Err=(|n|–1)for|n|>1and(|1/n|−1)forn<1 (14)

[SP] represents the concentration of *Sen* in which the regulatory “valve” is half opened, since k_reg([SP] = [Sen])_ = ½k_reg([SP]<<[Sen])_. From a biochemical perspective, [SP] reflects the binding strength of *Sen* to its transcription factor and indirectly to a gene promotor. [SP] values nearest to the mean of [Sen] for the three cellular states were preferred, as deviations of [SP] from this span of concentrations might be less chemically meaningful. We calculated this deviation using (15).


$$\mathrm{SP}\_\mathrm{Err}=\sum_{j=1}^{8}\frac{\left|[SP]-{\left[Sen\right]}_{mean}\right|}{{\left[Sen\right]}_{mean}}$$ (15)

For the remaining 2 cases, the sums of n_Err and SP_Err were similar and near the top scores for all 146 cases examined. These top cases, defined as the “best” cellular regulatory mechanisms for surviving all filters, are listed in Table 6.

**Table 6:** Autoregulatory parameters. The order of vertical entries within a column is for CRM cases 1 (left) and 2 (right). A single entry indicates the same value for both cases.

Parameter	*k*_*23*_	*k*_*cia*_	*k*_*vac*_	*k*_*mit*_	*k*_*cyt*_	*k*_*O2*_	*k*_*mp*_
*Sen*	*FC; FC*	*FM; FM*	*FC; FC*	*FS; FS*	*FC; F2*	*FS; FS*	*FM; FM*
n	0.249	-0.0388	0.680	-0.022	-1.00; -0.159	0.0135	0.0587
[SP]	15.1	0.992	14.0	0.992	8.74; 7.91	258	225
*k*_*reg*_	0.294	5.51	2.89	169	2.16; 16.6	95.4	0.931
*k*_*unreg*_	0	0.418	0.160	0.539	2.63; 2.63	0	0.00106

## Results

### Characteristics of the selected CRMs

The top two CRMs exhibited remarkably similar transition plots, so we selected one (Case 1) for highlighting. The regulatory structure of Case 1 is illustrated in Fig 1, lower panel. Both best cases had component FC (the labile Fe^II^ pool in the cytosol) as the sensor for the *k*_*vac*_ and *k*_*23*_ reactions, operating in feedforward regulation. Thus, both the import of vacuolar Fe^II^ from the cytosol and its oxidation to Fe^III^ (i.e. [F3]) are predicted to be feedforward-regulated by the concentration of the cytosolic labile iron pool. Informally, this makes sense because the vacuoles store excess cellular iron, and the “gate” allowing iron to flow into them should open when cytosol iron levels become too high. The setpoint concentrations for both relationships were nearly identical, suggesting the same regulatory event. That cells might regulate the import AND oxidation of vacuolar Fe^II^ has not been suggested in the literature; whether this occurs in real cells requires further investigation.

Both cases predicted that the rate of cytosolic iron import into mitochondria is feedback-regulated by FS, with setpoint concentrations significantly lower than the range of [FS] for the three cellular states. This is a fast reaction in which the unregulated rate is insignificant.

Both cases predicted that the rate of nanoparticle formation in mitochondria is feedforward-regulated by FM, the Fe^II^ pool in mitochondria. This implies that excessive concentrations of FM in the matrix stimulates nanoparticle formation. The setpoint concentration for this regulation was in the vicinity of the FM concentrations for the three cellular states. Again, the unregulated rate-constant was insignificant.

There was disagreement as to the sensor that feedback-regulates the rate of iron import into the cell; Case 1 predicts FC while Case 2 predicts F2. Iron import into real cells through the Fet3/Ftr1/Fre1 high-affinity system on the plasma membrane is feedback-regulated (along with other genes of the iron-regulon) by the transcription factors Aft1/2, whose DNA-binding activity is controlled by the activity level of ISC assembly in mitochondria. In our model, this corresponds to having FS be the sensor.

The remaining two regulatable reactions of the model, including the generation of CIA from the labile iron pool and the transport of O2 from the cell exterior to mitochondria, are more difficult to rationalize from a chemical perspective since little is known as to how (or if) these processes are regulated. Both cases predict that FM feedback-regulates *k*_*cia*_ and that FS feedforward-regulates *k*_*O2*_. Both predictions require experimental verification.

### Steady-state transitions

**Fig 3:**
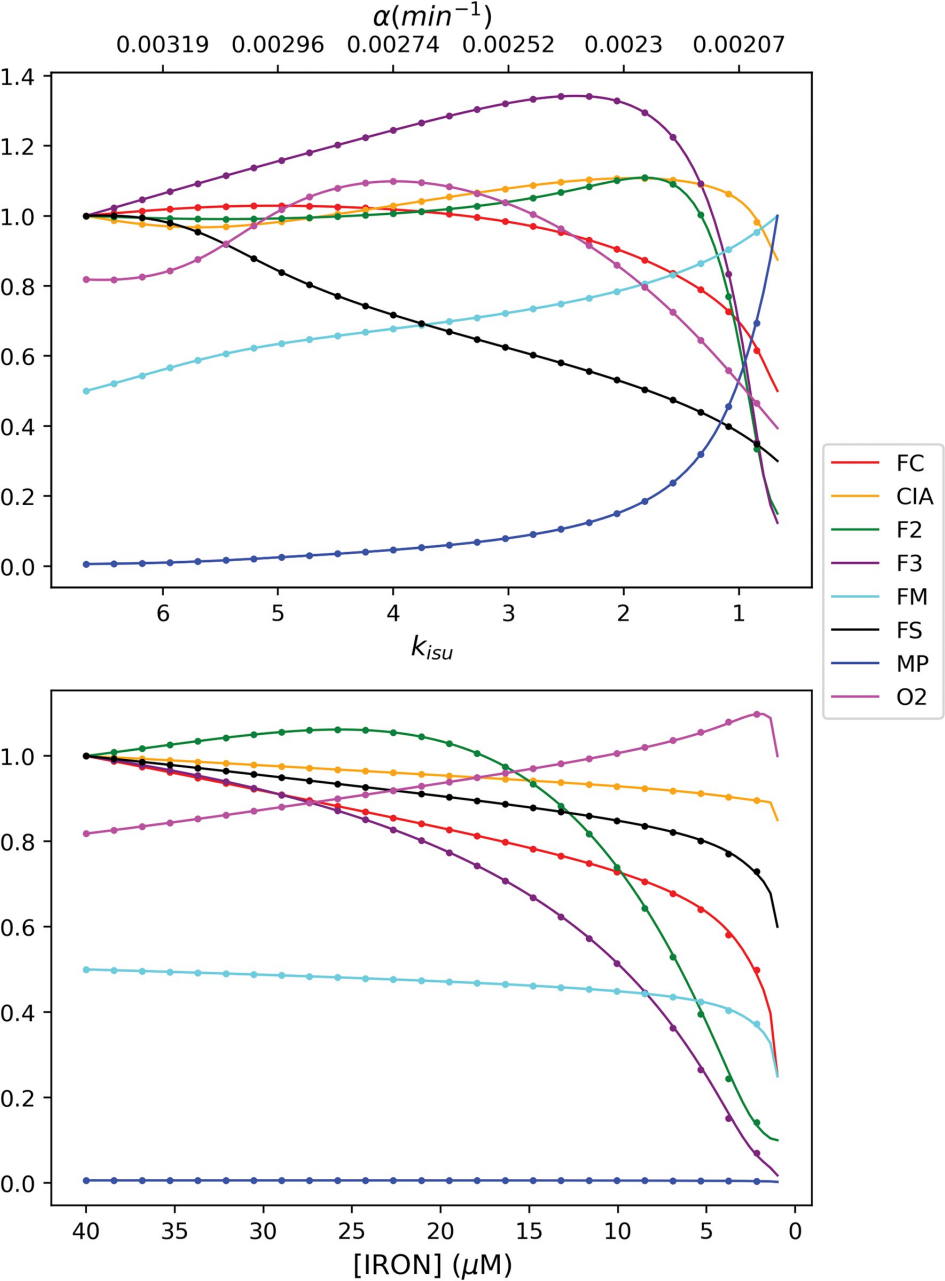
Normalized steady-state concentrations of model components during the transformation W → Y (top panel) and W → D (bottom panel). The following plots represent the steady state transition for the 2 cases. CRM 1 is represented by a solid line while CRM 2 is the dotted line.

Fig 3 reveals how the steady-state system, employing Cases 1 and 2 autoregulation, transitioned from W → Y (upper panel) and from W → D (lower panel). Plots were similar regardless of which top CRM case was assumed. For the W → Y conversion, [FS] declined smoothly and gradually (black line) as *k*_*isu*_ declined from 6.6 → 0.66 μM/min. This was expected given that FS is the product of the *k*_*isu*_ reaction. There were remarkably few dramatic changes in the concentration of other components until *k*_*isu*_ declined to ~ 2 μM/min. Thereafter, mitochondrial nanoparticles (MP) increased dramatically and vacuolar iron (F2 + F3) decreased dramatically. Fernandez et al observed similar behavior experimentally by examining Mössbauer spectra of cells as the concentration of frataxin was lowered [20]. They found that vacuolar iron empties *earlier* in the transition than MP accumulates.

The behavior of the steady-state system as it transitioned from iron-replete to iron-deficient conditions (Fig 3, lower panel) was similar to what is observed experimentally. As the *in silico* cell becomes increasingly iron deficient (left to right in the plot), vacuoles, which store iron under iron-replete conditions, empty their iron. This allows the concentration of other iron species to remain relatively unchanged until extreme iron-deficient conditions (e.g. [IRON] < ca. 1 μM) are attained. This buffering effect is important as it allows the cell to operate normally under a wide range of nutrient iron concentrations. O2 concentrations gradually increased as the cell became more iron-deficient because the rate of respiration slowly declined. Encouragingly, the CIA concentration remained relatively stable even under relatively extreme iron-deficient conditions. One group of Fe proteins symbolized by the CIA is non-mitochondrial ISC proteins in the nucleus. Nuclear ISC-containing proteins are perhaps the most essential iron centers in the cell, and so their concentrations would be expected to be the most resistant to decline under Fe-deficient conditions.

### Time-dependent Transitions

**Fig 4:**
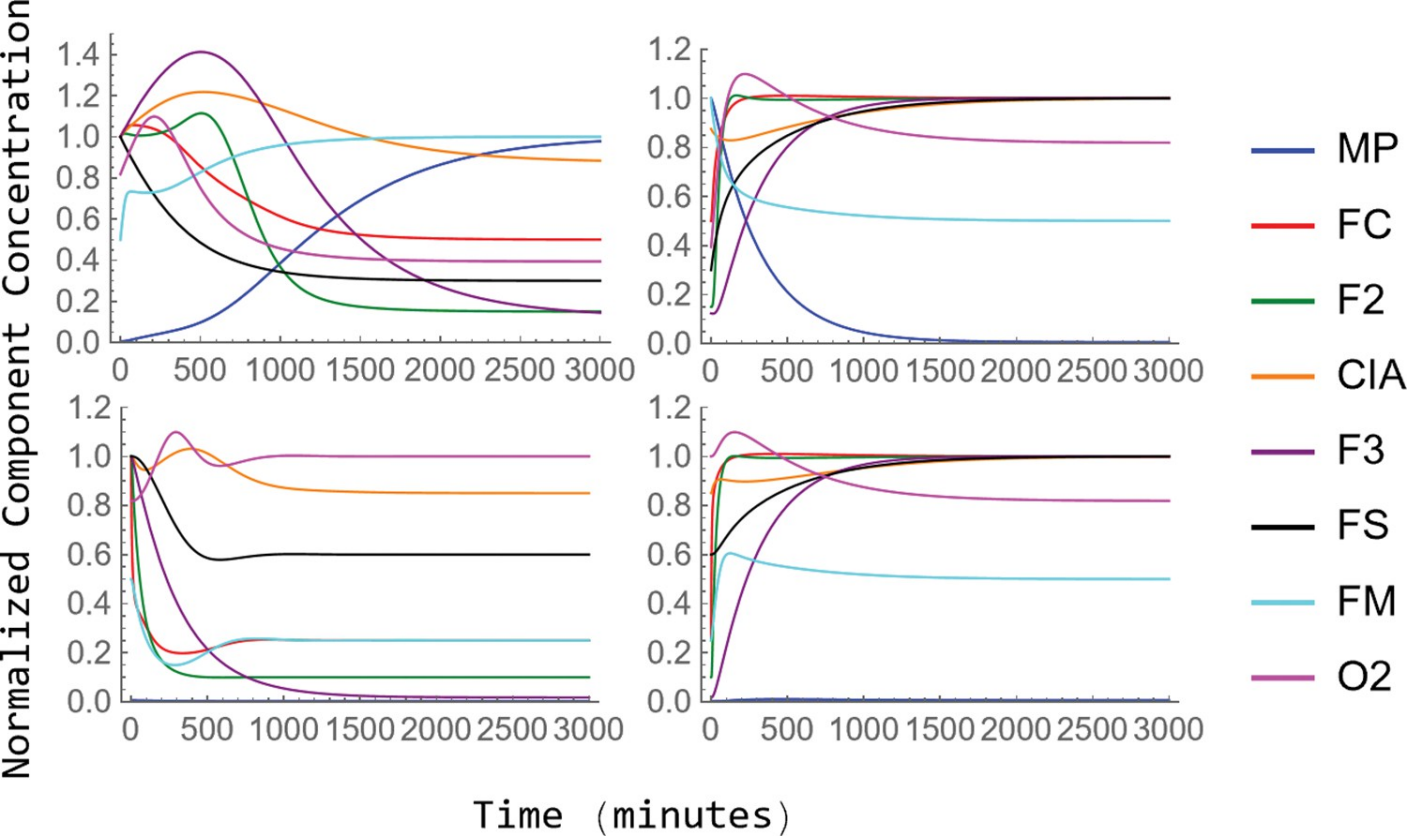
Normalized time-dependent concentrations of model components as the system transitions from W → Y and D → W (left side) and reverse (right side). Left two panels: W → Y (top) and W → D (bottom); Right two panels: Y → W (top) and D → W (bottom).

These plots for the W → Y and W → D transitions are given in Fig 4, top and bottom left panels, respectively. The healthy-to-diseased transition, stimulated by an abrupt 10× decline of *k*_*isu*_ (and reduction in α_cell_), required ca. 3000 min (2 days) which corresponded to ~ 9 doublings. The primary event led to a slow decline of [FS] along with faster transient increases in virtually all other components (except MP). Then, all these species gradually declined as [MP] increased. This choreography is predictive because the kinetics of the transition has not been investigated experimentally. Interestingly, the reverse transition, Y → W, was not the reverse of the forward process. In this case, all components returned to W concentrations faster, in ca. 1500 min, probably because the cell would be growing faster.

The W → D transition was faster (complete in ca. 1500 min) and showed the expected changes, including an emptying of vacuoles and a precipitous decline of FC and FM. These were followed by a more gradual and limited decline of [FS] and [CIA]. Thus, changes in [FS] and [CIA] were buffered at the expense of vacuoles emptying their iron; this makes sense physiologically. Again, plots of the reverse process were not the reverse of the forward plots.

### Stability analysis

**Fig 5:**
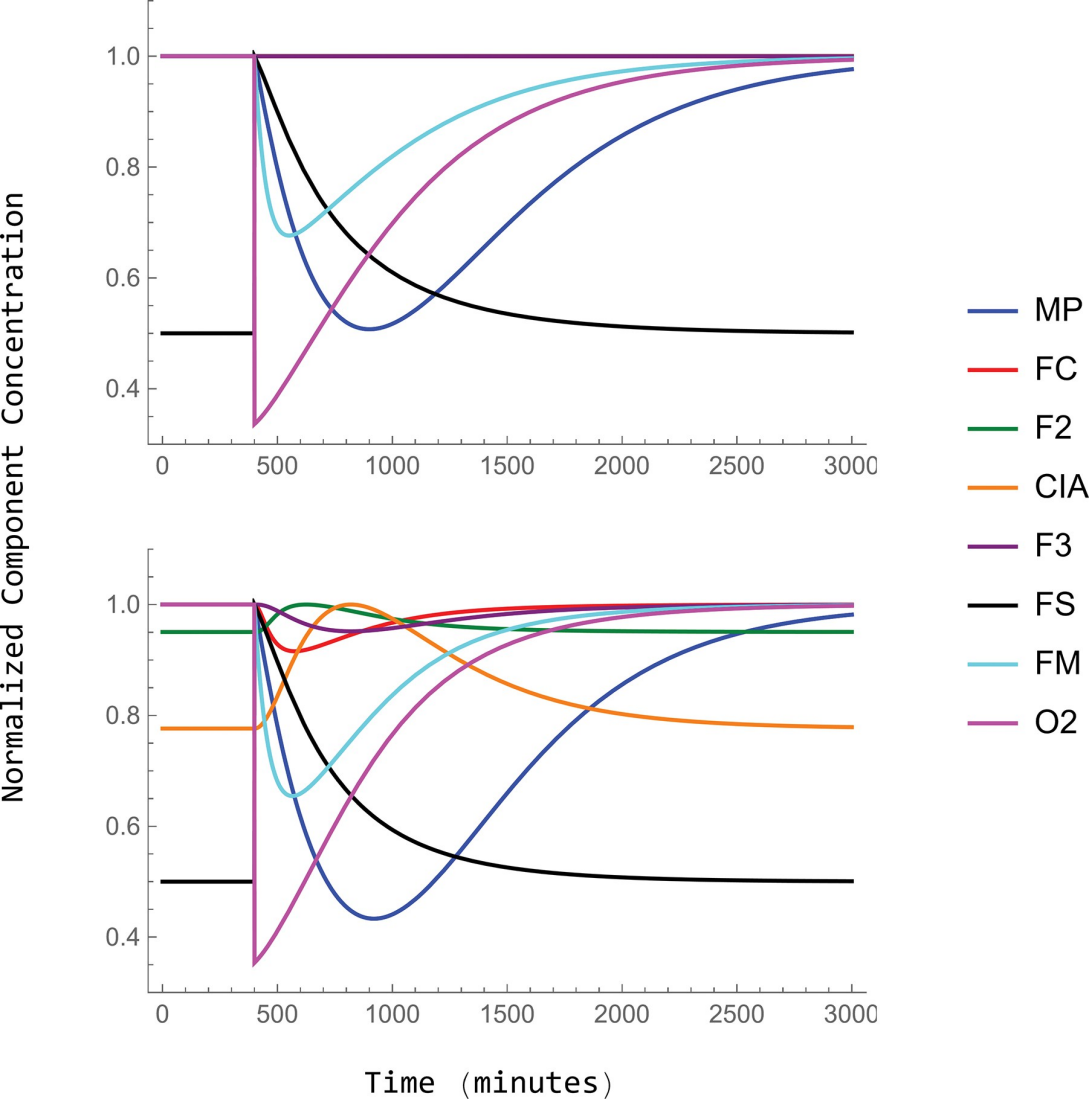
Perturbation and recovery. In this simulation, the system began in the W state with all components at their normalized steady-state concentrations except for [FS] which was normalized to 0.5. At t = 400 min, [FS] was doubled, and the system was allowed to recover in time. Top panel, without autoregulation; bottom panel, with Case 1 autoregulation.

The all-negative eigenvalues of the Jacobian matrix guarantee that the system recovers when the concentration of any component in the model is perturbed (within limits). To illustrate this, we abruptly doubled the concentration of [FS] for the W cell growing at steady-state. The steady-state system recovered regardless of whether the system was (Fig 5, lower panel) or was not (upper panel) autoregulated. In both cases, recovery required ~ 2400 min or ~ 11 doublings. Without autoregulation, only half of the components in the system were interrelated and thus perturbed; FC, F2, CIA, and F3 were unaffected by doubling [FS]. With Case 1 autoregulation included, *all* components of the system were impacted by the perturbation which seems more realistic. Immediately after [FS] was doubled, [FM], [MP], [FC], and [O2] declined and then recovered. [O2] declined majorly and instantly, due to the increased respiratory activity caused by doubling [FS], which in turn prevented O2 from entering mitochondria. [F3] declined as well but after a slight delay. [CIA] and [F2] both increased initially, and then recovered in accordance with the autoregulation of these components.

The eigenvalues obtained by the ***J*** matrix for the 3 states represent apparent first-order decay constants ($k_{i}\propto e^{\lambda_{i}t}$) for the associated component returning from a perturbed value. The eigenvalue for the recovery of component [FC] was orders-of-magnitude greater than for any other component (Table 5). This implies that the recovery from a perturbation in [FC] should be far faster than for other components. This makes sense because [FC] (the cytosolic labile iron pool) increases rapidly as nutrient iron enters cells, and it also decreases rapidly as FC iron is distributed into mitochondria, vacuoles, and cytosolic and nuclear proteins. In contrast, terminal components such as MP or F3 recovered far slower from a perturbation, as they relied solely on dilution to lower their concentrations.

### Sensitivity analysis

The sensitivity of the system in each cellular state was evaluated by determining the effect of changing each *k*_*obs*_ of the system on the steady-state concentration of each component *C*_*i*_. The magnitude of each *k*_*obs*_, augmented by a Heaviside function, was multiplied by a scale factor *h* with values ranging from 0.5 to 2.0. This factor was increased in increments of *j* = 0.01. The effect of “jiggling” a selected *k*_*obs*_ in this way on the steady-state concentration of a selected component *C*_*i*_ was determined by calculating the slope of a function designated *G*_*i*_*(h)*. For each value of *h*, this function returned the percent change in the steady-state concentration of *C*_*i*_. The greater the percent change at *h* = 1 (i.e. the greater the slope of *G*_*i*_*(h = 1)*), the more sensitive that *k*_*obs*_ was considered. For a given *k*_*obs*_, the process was repeated for each *C*_*i*_, and the resulting normalized slopes were summed and then averaged. A larger slope indicated a greater sensitivity of that *k*_*obs*_. The sensitivity of a *k*_*obs*_ was calculated as

$$\mathrm{Sensitivity}\;\mathrm{of}\;a\;k_{obs}=\frac{1}{8}\sum_{i=1}^{8}\left|\frac{\left(\frac{G_{i}(h-2j)-8G_{i}(h-j)+8G_{i}(h+j)+G_{i}(h+2j)}{12j}\right)}{\left[C_{i}\right]}\right|$$ (16)

by a finite difference approximating the derivative with a Taylor’s series [40][41]. Table 7 indicates the sensitivity of each *k*_*obs*_ for each cellular state. Assuming Case 1 (Table 6), *k*_*O2*_ was the most sensitive for all three cellular states, indicating the great importance of the rate of oxygen entering the system. The rate of iron entering mitochondria (*k*_*mit*_) was the second-most sensitive. In the model, the reactions involving iron and oxygen within mitochondria are clearly critical in controlling overall behavior. The least sensitive rate-constant, again for all three cellular states, was *k*_*23*_ which reflects the oxidation of *F2* to *F3* in vacuoles. Also interesting is that the Y state was overall least sensitive to any change in rate (by summing up all the sensitivity numbers); one could view the healthy W state as “spiraling down” and becoming “locked into” the diseased Y state.

**Table 7:** Sensitivity analysis. Sensitivities were calculated according to Eq (16). They represent the sum of percentage changes in the concentration of each component in the model when the indicated rate constant was “jiggled”.

Rate constant	W	Y	D
*k*_*O2*_	59	40	47
*k*_*mit*_	26	1.7	11
*k*_*cyt*_	23	1.9	21
*k*_*vac*_	16	0.79	2.6
*k*_*cia*_	3.0	0.33	11
*k*_*mp*_	2.5	0.87	1.6
*k*_*23*_	0.15	0.59	0.74

### Predicting cellular states

**Fig 6:**
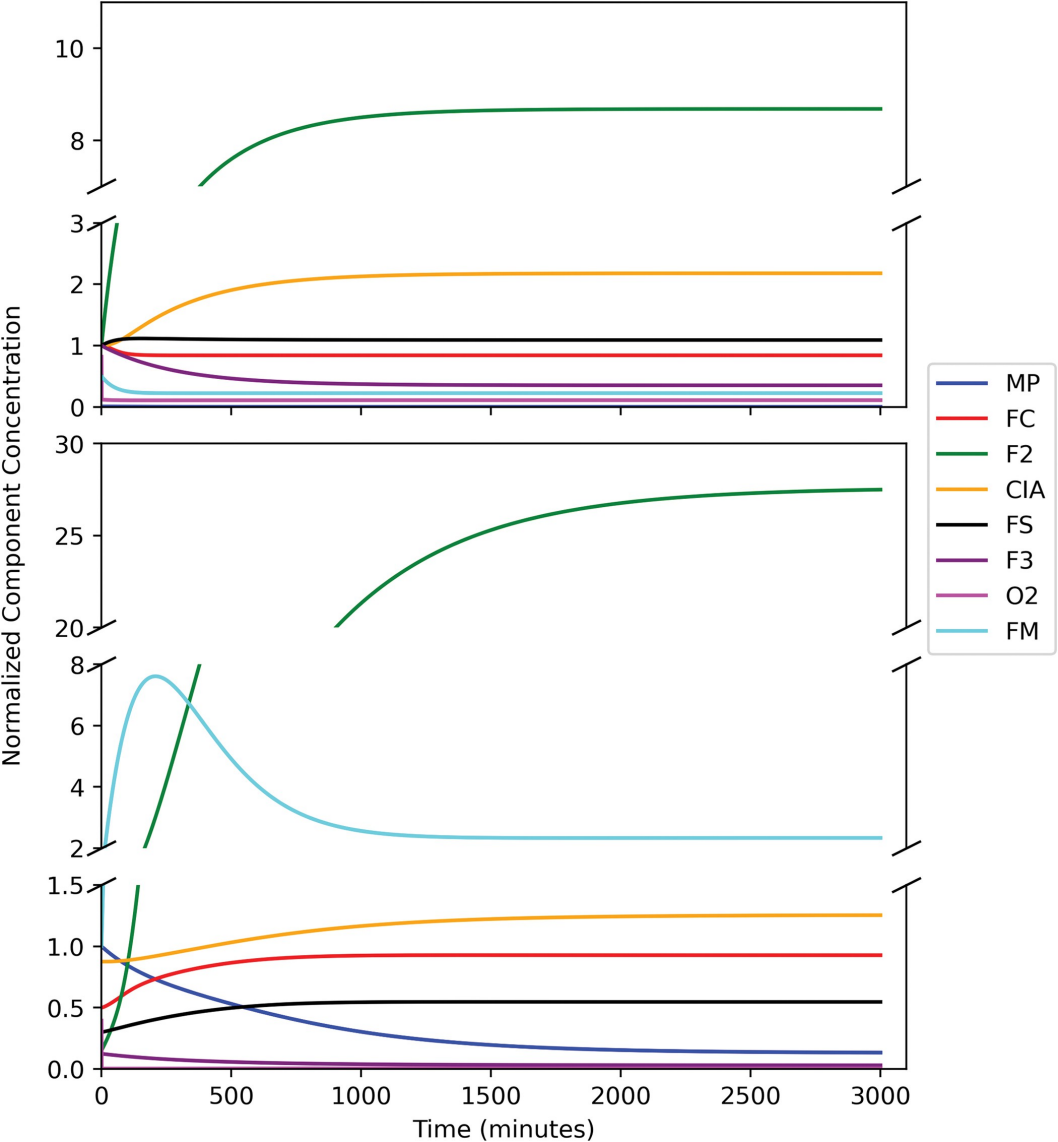
Predicting the effect of hypoxia. The system began in the W (left panel) and Y (right panel) states. At t = 0, the concentration of O2 was reduced from 100 μM to 25 μM (top) or to 1 μM (bottom). This caused the system to transition from W → H_W_ (top) and Y →H_Y_ (bottom).

Finally, we used our model to predict the iron content of WT and Yfh1-deficient yeast cells grown under hypoxic conditions, called the H_W_ and H_Y_ states (Fig 6, top and bottom panels), respectively. The model was not trained on these states so our results represent true predictions. The H_W_ state was obtained simply by reducing [OXYGEN] from 100 μM in the W state to 25 μM; the H_Y_ state was obtained likewise from the Y state, except that [OXYGEN] was reduced to 1 μM. In this latter case, [MP] and [F3] declined majorly while [F2] (Fig 6, green line) became dominant. Indeed, Mössbauer spectra of the H states are dominated by a nonheme high-spin Fe^II^ quadrupole doublet, and nanoparticles are absent [20]. Importantly, the steady-state concentration of [FS] in the H_Y_ state (Fig 6, black line, lower panel) is predicted in our simulations to be ~ 5× higher than in the Y state, and comparable to [FS] concentrations in the W and D states. A similar recovery under hypoxic conditions was observed experimentally [20]. Moreover, raising frataxin-deficient mice under hypobaric conditions attenuated disease progression [42].

## Discussion

In this study, we solved a coarse-grain ODE-based biochemical kinetic model operating within growing yeast cells. The model describes how iron is trafficked and regulated in eukaryotic cells. The system was solved to allow both dynamic (time-dependent) and steady-state simulations. To do this, we relied on steady-state concentrations for each component in each of the three considered cellular states (W, Y, and D). Component concentrations for these systems were assigned based on previously published Mössbauer spectroscopic results in combination with determinations of iron concentrations in whole cells, mitochondria, vacuoles, and cytosol. The model also employed experimentally determined growth rates for cells.

Like most ODE-based biochemical models, sufficient kinetic information was unavailable to solve the system rigorously and uniquely, whereas substantial concentration data were available. Relying on concentrations of cellular components increasingly makes sense because such quantitative concentration determinations are becoming increasingly available due to mass-spectrometry-based proteomic and metabolomics studies. In contrast, determining kinetic parameters experimentally for individual biochemical reactions remain an arduous task.

The current model evolved from earlier versions [19, 20] and uses an improved method of optimization. Both earlier versions were solved only at steady-state; here time-dependent dynamic states were also obtained. The regulatory mechanisms assumed in earlier versions were designed by the modeler as needed to generate steady-state concentrations that approximated experimental results by adjusting kinetic parameters at will. In optimizing the current model, steady-state concentrations were assigned to be most consistent with experimental results, and then the set of kinetic parameters that would generate those concentrations exactly or nearly so was calculated. This was a major advance.

The traditional notion of solving transient dynamical systems is to assign all system parameters and then perform numerical simulations to observe the evolution of the state variables including approximating their steady state values. For dynamical systems corresponding to metabolic networks, the system parameters correspond to reaction rates while the state variables are metabolite concentrations. Assigning values to all the parameters needed to determine the reaction rates as functions of the state variables is a formidable task. The approach taken here capitalized on the specific structure of the dynamical system corresponding to a metabolic network within an exponentially growing cell which included amongst the system reactions, dilution terms for the state variables. The reaction rates are determined by consideration of the steady state counterpart to (2) in which the right-hand-side is set to the zero-vector giving a homogeneous, linear algebraic system with the vector of reaction rates as the unknown. By organizing the stoichiometric matrix so that the respiration rate *R*_*res*_ and the eight dilution rates are the final nine entries in the reaction rate vector, the RREF in (3) gives the respiration rate and the eight dilution rates as the nine degrees of freedom in the general solution to (3) with the other eight reaction rates being linear functions of those nine degrees of freedom. Reasonable estimates for those nine free rates were measured experimentally or gleaned from the literature; the remaining reaction rates were then calculated from the relations (4). This allowed the entire steady-state system to be solved for each cellular state considered. The stability of the resulting systems was demonstrated by constructing the Jacobian matrix and solving for its eigenvalues (Appendix B in S1 Text and Table 5).

For the most part, each cellular state used different steady-state concentrations as well as a unique set of rate-constants. The next challenge was to have the model transition from the wild-type iron-replete state (W) to a diseased state characteristic of Friedreich’s Ataxia (Y) as well as to an iron-deficient wild-type state (D). Transitions were triggered by simple primary events initiated external to the model. The W → Y transition was triggered by a decline in the rate-constant associated with the assembly of mitochondrial ISC coupled to a decline in the growth rate of the cell. This mirrored the primary cause in the development of Friedreich’s Ataxia. The W → D transition was triggered by lowering the nutrient iron concentration.

Without regulation, the *in silico* cell’s response to these primary events was insufficient to successfully transition from W to Y or D states, because the set of rate-constants used for each state were (generally) different from each other. In essence, we had “tied-down the two ends” of the desired transitions (W → Y and W → D) but needed to solve the system between those ends. We reasoned that transitioning must involve the gradual shifting of rate-constants, from the set that defined the initial W state to those which defined the final Y or D states. Adjusting these rate-constants manually would be artificial (nonbiological), so a strategy was developed to do this automatically in response to the primary events.

The strategy involved autoregulation which would exclusively use model components as sensed species (i.e. sensors) in regulation. The concentration of a designated sensor, relative to a set-point concentration, could regulate the rate of a reaction. Real cells do this to maintain homeostasis, making autoregulation superior to externally/manually controlled mechanisms.

The reactions of the model did not involve enzymes explicitly, but we reasoned that rate-constants for a given reaction could be viewed as reflecting the expression level of an implicit enzyme. Thus, shifts in rate-constants from one state to another were viewed as reflecting shifts in gene expression levels during transition.

The actual biochemical mechanisms by which gene expression levels are controlled were either too complicated to be employed in autoregulation, or they were unknown. Thus, we decided to augment every regulatable reaction using soft Heaviside functions as surrogate regulatory systems. For each such reaction, we needed to identity which component would best serve as sensor in shifting the set of rate constants appropriately. This would constitute the best CRM.

To do this, we first assumed all possible combinations of sensed species for the group of regulatable reactions. We then applied six criteria, called *uniqueness*, *trending*, *targeting*, *wandering*, *smoothness*, an *n/SP-reasonableness* to filter or select the best CRMs, similar to a genetic screen. In the end, two CRM cases all had similar “fitness”, and one was highlighted in constructing transitional plots. We caution that applying the same strategy for selecting viable autoregulatory mechanisms will become increasing difficult computationally as the complexity of models increases.

The problems we faced are the norm for kinetic biochemical models within the field of cell biology; thus, our methods might be useful in solving other such models. The quality and quantity of available kinetic information, and the correctness of the proposed mechanism and rate-laws ultimately limit the reliability and predictive power of simulations. However, there are many situations within the field of cell biology where simulating even qualitative or semi-quantitative behaviors would provide new insights and advance the field. In such cases, a more rigorous mathematical analysis, as given here, could constrain possible solutions and render models more reliable.

One advantage of mathematical models is that they are fully transparent in terms of assumptions, the parameters required and values used, the sensitivity of that information to the behavior of the model, etc. Allowing the entire system to be open for inspection and criticism will advance our understanding of the modeled process to a far greater extent than can be gleaned from cartoon mechanisms (e.g. Fig 1 alone) which are commonly included in papers and reviews.

Once a mathematical model is operational, an iterative process of making predictions, testing and modifying can commence. As more reliable information becomes available, models will also become more reliable and possess greater predictive power. Ultimately, a model with predictive power could be used to understand (on a biochemical mechanistic level) the effect of a genetic mutation and consequent disease formation. Such models could also be used to evaluate the effect of various therapies and treatments for diseases. This would be highly useful to researchers, clinicians–and most importantly, patients.

## Supporting information

S1 Text [21, 33, 43–48]. Appendix A. How *R*_*res*_ = 9090 μM/min was selected; Appendix B. Jacobian matrices for the W, Y, and D states; Appendix C, Concentration estimates in Table 2; Fig A., example of a CRM that failed the Targeting filter; Fig B., example of a CRM that failed the Wandering filter; Fig C., example of a CRM that failed the Smoothness filter.
(DOCX)
