# Supplementary material for: A kinetic model of iron trafficking in growing Saccharomyces cerevisiae cells; applying mathematical methods to minimize the problem of sparse data and generate viable autoregulatory mechanisms
Source: PLoS Comput Biol. 2023 Dec 19;19(12):e1011701. doi: 10.1371/journal.pcbi.1011701 (PMC10729996; doi:10.1371/journal.pcbi.1011701)

**Supplemental Information**

**Title:** A kinetic model of iron trafficking in growing *Saccharomyces cerevisiae* cells; applying mathematical methods to minimize the problem of sparse data and generate viable autoregulatory mechanisms

**Authors:** Shantanu Thorat, Jay R. Walton, and Paul A. Lindahl

Appendix A. How *R_res_* = 9090 µM/min was selected.

Appendix B. Jacobian matrices for the W, Y, and D states.

Appendix C. Estimation of concentrations in Table 2.

Fig A Example of a CRM that failed the Targeting filter.

Fig B. Example of a CRM that failed the Wandering filter.

Fig C. Example of a CRM that failed the Smoothness filter.

**Appendix A. How *R_res_* = 9090 µM/min was selected:** Popel (2003) [38] reported a “mitochondrial-based maximum O_2_ consumption” rate of 5·10^-3^ ml O_2_ ml^-1^ sec^-1^. This value was based on the maximum mitochondrial respiration rate of 0.1 ml O_2_ (ml mito)^-1^ sec^-1^ and a volume fraction of mitochondria in vascular cells of approximately 5%. We assumed that ml^-1^ in the rate expression meant “per ml of mitochondria”. To convert ml of O_2_ to moles of O_2_, we used the ideal gas law n = (1 atm)(5×10^-6^L)/0.082 L⋅atm/mole⋅K)(310 K) = 1.97×10^-7^ moles O_2_. Thus, the rate became1.97×10^-7^ moles O_2_ per mL mitochondria per sec. The volume of mitochondria in a yeast cell was assumed to be 2.14×10^-12^ mL, so the O_2_ consumption rate became 4.21×10^-19^ moles O_2_ per sec. This is the O_2_ consumption rate within an entire yeast cell, which has a volume of 42×10^-15^ L, so the O_2_ consumption rate in terms of concentration of O_2_, became 1.002×10^-5^ M O_2_/ sec or 601 µM/min (cell concentration).

We had difficulty distinguishing this rate from the maximum mitochondrial respiration rate given above (0.1 mL O_2_ per mL mito per sec). Applying the ideal gas law gave 3.94×10^-6^ moles O_2_, so that rate became 3.94×10^-6^ moles O_2_ per mL mitochondria per sec. Multiplying by the estimated total volume of mitochondria in the cell gave 8.4185×10^-18^ moles of O_2_ per sec per cell. Dividing by the volume of the cell afforded 2.004×10^-4^ M O_2_ per sec. or 12,000 µM/min O_2_ consumed (local concentration in the mitochondria). If we multiply by 5% (the approximate percentage of cell volume due to mitochondria), we get 601 µM/min, the value obtained above. This suggested that 601 µM/min is the cellular consumption rate and *R_res_* = 12,000 µM/min O_2_ is the local rate of O_2_ consumption within respiring mitochondria. For the model, we initially rounded this rate to 10,000 µM/min. However, the value needed to be adjusted slightly (to 9090) such that all eigenvalues in the Jacobian matrix were negative.

**Appendix B: Jacobian matrices for the W, Y, and D states.** The top three are without regulation functions; the bottom three are with CRM Case 1 included.

W Matrix


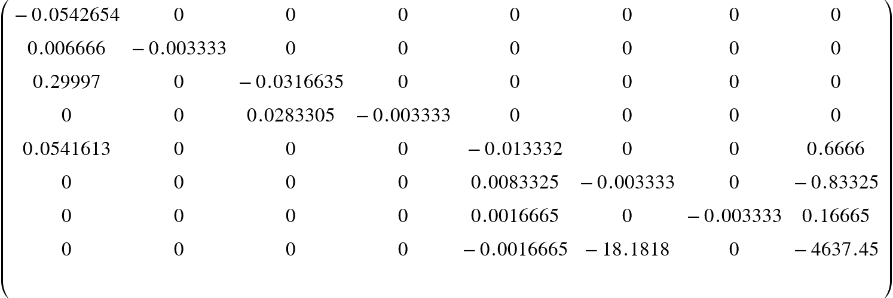


Y Matrix


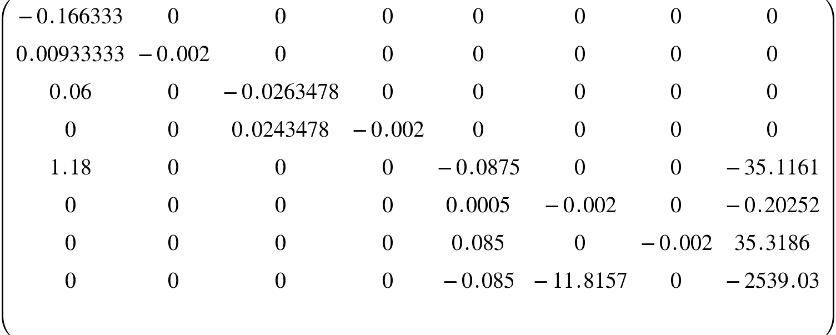


D Matrix


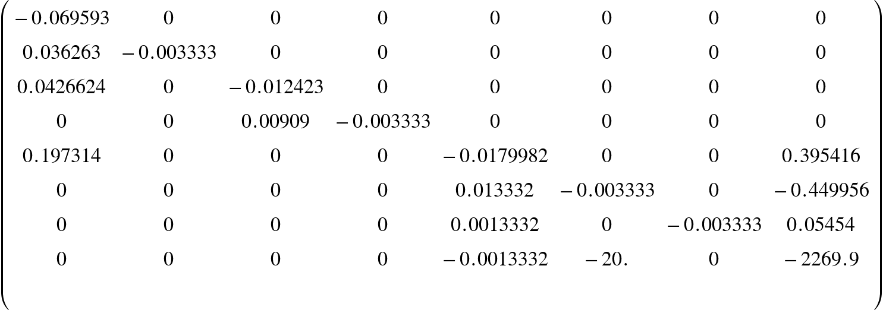


With CRM 1 reg functions added:


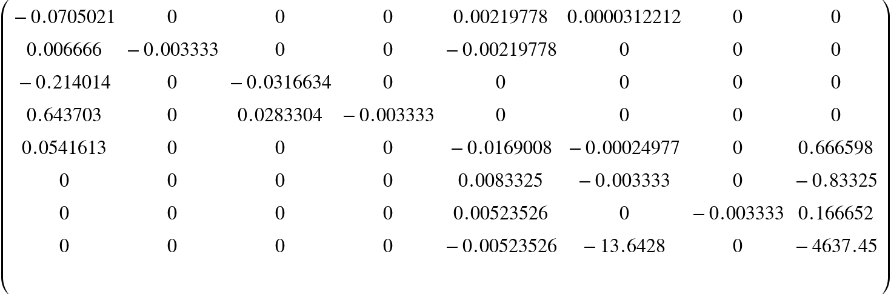
W Matrix

Y Matrix


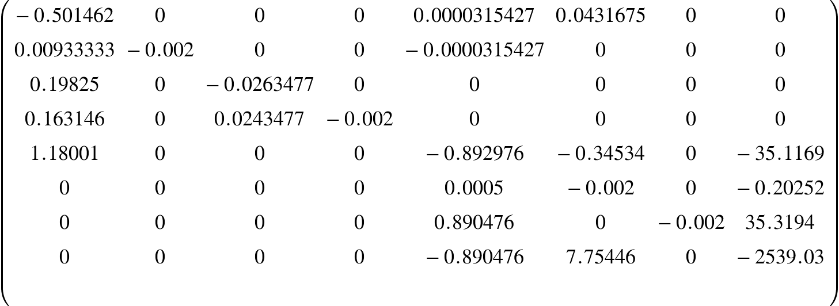


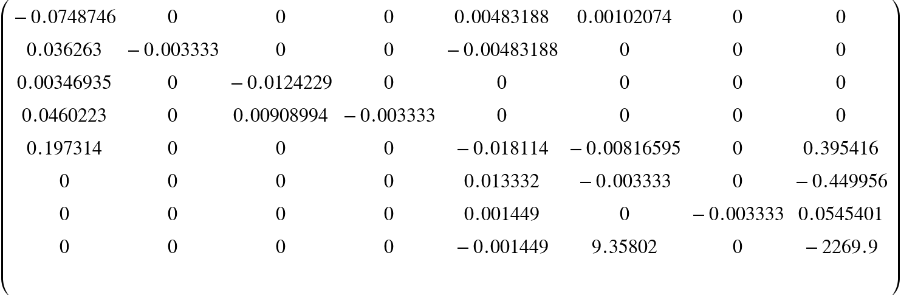
D Matrix

**Appendix C. Concentration estimates in Table 2**

Concentrations given in Table 2 are the corresponding author’s best estimates for each modeling component in *S. cerevisiae* cells in the W, Y, and D states. These numbers should be viewed as *estimates*, not strict experimental determinations, due to the insufficient amount of relevant data available and an unusually wide range of values published. Determining concentrations of biochemicals *within growing cells* is far less precise than doing so in a solution containing a single biochemical. Concentrations in growing *S. cerevisiae* cells depend on the strain used, the growth rate at the time of harvesting, the media used, whether cells were fermenting or respiring, the concentration and type of iron used to supplement the growth media, and whether the cells were grown under aerobic vs hypoxic conditions, terms which themselves are ill-defined. The concentrations of cellular components likely depend on the method by which the cell wall is removed and how the sample is treated prior to measurement (fresh, stored, or freeze/thawed?).

Ideally, for this computational model, we assumed strain W303 grown in minimal media containing either 1 µM or 40 µM Fe^III^ citrate under respiring conditions (i.e. growth on glycerol/ethanol or lactate as carbon sources) and under aerobic conditions (vigorous swirling in shaker flasks). Cell walls would have been removed by zymolyase treatment and concentration determinations would have been made fresh, immediately thereafter. It’s unlikely that all concentration determinations considered in developing the table were obtained with cells prepared under those conditions.

Another important consideration is that the concentrations in the table are given in *absolute* terms, in units of µM, rather than as a percentage of a WT level or as a ratio of the same parameter in one state vs. another. Determinations of absolute concentrations are more difficult because they involve keeping track of fold-dilutions during each step of the assay as well as knowing (or estimating) the packing efficiency of cells or organelles during centrifugation steps. Few researchers in this field publish absolute concentrations.

***Conservation of matter relationships:*** Our analysis relied on accounting relationships based on the conservation of matter. The following relationship relates the whole-cell iron concentration to the concentration of all iron components in the model and the fractional volume for each compartment.

$$[Fe_{cell}]=f_{cyt}[CIA]+f_{vac}[F2]+f_{vac}[F3]+f_{cyt}[FC]+f_{mit}[FM]+f_{mit}[FS]+F_{mit}[MP]$$

Fractional volumes in this model were assumed to be 0.8, 0.1, and 0.1 for cytosol, mitochondria and vacuoles respectively. Estimates of fractional volumes in yeast cells were recently made [43] but that study included two additional cellular compartments, namely the nucleus and endoplasmic reticulum. The simpler estimates used here, in which the cell has been divided into just 3 compartments, are reasonable for respiring cells as long as the “cytosol” in the model is defined to include these other two compartments. Another conservation-of-matter relationship,

$$[Fe_{mit}]=[FM]+[FS]+[MP]$$

relates the concentration of iron in isolated mitochondria to the component concentrations. Similar relationships for vacuoles and cytosol

$[Fe_{vac}]=[F2]+[F3]$ and $[Fe_{cyt}]=[FC]+[CIA]$

were also considered but were less useful as fewer determinations of the iron concentration of vacuoles and cytosol have been reported than for mitochondria or whole cells. These equations constrained values for each component in the table. Ultimately, concentrations were adjusted (or “jiggled”) from direct determinations to make them compatible with these relationships. This procedure involved judging the reliability of reported values and weighing some more heavily than others. However, efforts were made to minimize such subjective influences.

***Connection between Mössbauer spectra and modeling components:*** Much of the experimental data used for component concentrations were obtained from Mössbauer spectra. Whole-cell spectra (5 K, 0.05 T) were typically decomposed into 5 species, including nonheme high spin (NHHS) Fe^II^, NHHS Fe^III^, the central quadrupole doublet (CD), nanoparticles, and heme Fe^II^. The following relationships between Mössbauer spectral features in whole-cell samples and model components were assumed.

The concentration of model component FS was defined to be the sum of the iron-sulfur clusters [ISC] and hemes [Heme] in mitochondria. Both ISCs and hemes are generated using the same pool of mitochondrial Fe^II^ (FM in the model). The concentration of each Mössbauer species was obtained by multiplying the percent absorption for that spectral feature by the concentration of iron in the cell ([Fe_cell_]). Then these relationships were applied. All concentrations are in µM.

***Whole-cell iron concentrations:*** We mainly relied on values reported by Holmes-Hampton et al. [22] for whole-cell concentrations for the W and D states, even though they were obtained using fermenting cells and at different Fe concentrations in the media than assumed. For the W state, whole-cell concentrations of 395, 470, 440, and 450 µM were reported for cells grown on minimal media supplemented with 10, 100, 1000, and 10,000 µM Fe. The average concentration of 440 µM was reasonably near to the computed value of 505 µM obtained using the concentrations listed in Table 2:

$$[Fe_{cell}]=0.8[80]+0.1[200]+0.1[3400]+0.8[20]+0.1[100]+0.1[500]+0.1[50]=505$$

We regard the 15% difference in the two numbers as acceptable. Also, a higher concentration would be expected for respiring (computational) vs. fermenting (experimental) cells.

For the D state, Holmes-Hampton et al. [22] reported 170 and 240 µM Fe for cells grown in minimal media on galactose and glucose, respectively, and containing the chelator bathophenanthroline sulfonate (BPS) but also supplemented with 1 µM ^57^Fe. These were not rigorously Fe-deficient conditions, but the added ^57^Fe was required to observe Mössbauer spectra. Under more severe Fe-deficient conditions, the whole cell concentration might be lower than 205 µM, the average of these numbers. Moore et al. [44] reported a concentration of 120 µM for cells grown with 25 µM BPS and 1 µM ^57^Fe in respiring growth media. In a recent bioinformatic study [43], the iron concentration of respiring yeast cells under iron-deficient conditions was calculated to be 123 µM. Using the values for the D state in Table 2, we computed 104 µM using the relationship

$$[Fe_{cell}]=0.8[68]+0.1[20]+0.1[60]+0.8[5]+0.1[50]+0.1[300]+0.1[20]=104$$

This value is acceptably near to the experimentally determined values for Fe-deficient cells.

For the Y state, we relied on the whole-cell iron concentration for Yfh1-deficient cells [20], namely 1780 µM.

$$[Fe_{cell}]=0.8[70]+0.1[30]+0.1[420]+0.8[10]+0.1[200]+0.1[150]+0.1[8500]=994$$

The computed concentration is admittedly lower than desired. A better match could have been made by assuming a higher concentration of nanoparticles but other constraints (see below) prevented us from assuming this.

Mössbauer spectra of the Y state cells included 8% (140 µM) due to high-spin Fe^III^ (F3 in the model). In the table, we estimate 420 µM for this component but this value is the concentration in isolated vacuoles. Since the fractional volume for vacuoles was 0.1, this would correspond to a cell concentration of 42 µM. A NHHS Fe^II^ spectral feature is also evident (see Figure 5, C and D of [20]), which we estimate at ~ 4% (70 µM). This feature includes contributions from [F2], [FC], and [FM]. The values in the table for these three components were 30, 10, and 200, respectively. With fractional volumes of 0.1, 0.8, and 0.1 respectively, the equivalent whole-cell concentration for the sum of these three components would be 31 µM; this number should be compared to the 70 µM just described. Substituting in values from the table into the whole-cell iron relationship gives

$$[Fe_{cell}]=0.8[70]+0.1[30]+0.1[420]+0.8[10]+0.1[200]+0.1[150]+0.1[8500]=994$$

For the Y state. The calculated whole-cell concentration was less than half of that reported, but adjusting these concentrations to correct this would cause the calculated Fe concentration of Yfh1-deficient mitochondrial to be too high. This is an example of how the values in the table often represent a compromise.

***Isolated Mitochondria iron concentrations:*** Mitochondria isolated from W cells contained 544, 710, 840, and 500 µM Fe (for cells grown on 10, 100, 1000, and 10,000 µM media iron) [22]. The average value (650 µM) was equivalent to the value calculated from the relationship

$$[Fe_{mit}]=[FM]+[FS]+[MP]=100+500+50=650$$

The equality of these two numbers is coincidental. Morales et al [26] reported Fe concentrations for mitochondria isolated from respiring, respirofermenting, and fermenting iron-replete cells, affording an average of 780 µM. Spectra could be decomposed as follows:

Central doublet = 45% (351 µM)

NHHS Fe^II^ = 8% (65 µM)

Fe^II^ hemes = 5% (39 µM)

Nanoparticles = 13% (99 µM)

We assumed the following relationships to connect MB spectral intensity to component concentrations.

Thus, the concentrations reported [26] imply that [FM] = 65 µM; [FS] = 351 + 39 = 390 µM; and [MP] = 99 µM. The corresponding values in the table were [FM] = 100; [FS] = 500; and [MP] = 50. Differences were less than a factor of 2.

For mitochondria in the D state, we considered the average concentration of Fe reported for mitochondria isolated from Fe-deficient cells [22], namely (300 + 480)/2 = 390 µM. Moore et al. [44] reported 430 µM for the iron concentration in mitochondria isolated from cells grown on 1 µM Fe. The value calculated using model component concentrations,

$$[Fe_{mit}]=[FM]+[FS]+[MP]=50+300+20=370$$

370 µM, was in reasonable agreement with these experimental determinations.

For the Y state, we relied on the iron concentration and Mössbauer spectra of mitochondria isolated from Yah1-deficinet cells [45] and from Atm1-deficient cells [25]. Doing so is acceptable because the spectral features of both mutants are essentially the same as that from Yfh1-deficient mitochondria – all are dominated by nanoparticles with a minor peak due to NHHS Fe^II^. Miao et al. [45] reported mitochondria concentrations of 7400 µM and Miao et al. [25] reported a mitochondrial Fe concentration of 8900 µM. The average of these two concentrations (8200 µM) should be compared to that calculated using the equation

$$[Fe_{mit}]=[FM]+[FS]+[MP]=200+150+8500=8850$$

namely 8850 µM Fe. We could match the values by adjusting down the [MP] concentration especially, but this would negatively impact the calculation above regarding the whole-cell iron concentration for the Y state. Thus, we were constrained to compromise in assigning concentrations.

***Mitochondrial Iron-sulfur clusters (FS):*** We selected values of 500, 150, and 300 µM for [FS] in W, Y, and D states respectively (Table 2). In addition to the considerations just given, we assumed that the concentration of [FS] in the D state should be somewhat less than in the W state and that [FS] in the Y state should be even less since frataxin is a component of the protein complex that catalyzes the assembly of ISCs, and the lack of frataxin should have major consequences in this regard. The more important issue was whether ***any*** ISCs are assembled in the Y state. This was difficult to evaluate under aerobic conditions because the doublet due to the nanoparticles overwhelms the spectra such that the central doublet is hidden. However, there are many indications that some ISC are assembled under Yfh1-deficient conditions. Miao et al. [45] found that aconitase and succinate dehydrogenase, two ISC-containing enzymes in Yah1-deficient mitochondria (equivalent to Yfh1-deficient mitochondria) had significant activities relative to WT mitochondria. Also, Fernandez et al [20] found that ISCs are generated in Yfh1-deficient cells grown under hypoxic conditions. Both imply that ISCs ***can*** be assembled in the absence of Yfh1, albeit at a slower rate (and with significant degradation under normoxic conditions). Consistent with that, Das et al [46] found that including frataxin increased ISC assembly activity by ca. 3-fold, similar to what we assumed for the ratio of [FS]_W_/[FS]_Y_ = 500/150 = 3.3.

***Vacuolar iron concentrations:*** These organelles have been isolated and characterized for their iron content [23]. Vacuoles were found to contain NHHS Fe^III^ similar to that which dominates whole-cell spectra of iron-replete cells. The absolute concentration of iron in the organelles was less than estimated from the percentage of iron in whole cell spectra, suggesting that most iron in the organelle leaches out during vacuolar isolation. In that study, vacuolar iron represented ~ 75% of Mössbauer spectral intensity of iron-replete cells. Likewise, Holmes-Hampton et al. [22] determined the percentage of spectral intensity due to NHHS Fe^III^ in whole cells grown on media containing different concentrations of iron. For Fe-deficient cells, little or no NHHS Fe^III^ was observed. For cells grown on 1, 10, 100, 1000, and 10,000 µM Fe, vacuolar iron represented 40%, 76%, 80%, 75%, and 84% of whole-cell spectral intensity. Thus, it would appear that vacuoles load iron gradually, and when ‘filled” represent ~ 75% of spectral intensity. Accordingly, if cells contained 505 µM Fe (as calculated in Table 2), then ~ 380 µM ought to be due to vacuoles. And if vacuoles have a fraction volume of 0.1, then the concentration of vacuolar iron should be ~ 3800 µM. This compares favorably with the value of 3400 µM for F3 and F2 = 200 µM in Table 2. Under Fe-deficient conditions, vacuoles will be largely devoid of iron. In the table we assumed 60 µM for F3 and 20 µM for F2, corresponding to ~ 8% of cellular iron and also of spectral intensity. At that intensity, it would be difficult to distinguish the magnetic features of NHHS Fe^III^ from the spectral baseline.

***The CIA iron concentration:*** The CIA refers to the Cytosolic Iron sulfur cluster Assembly complex, which in real cells is used to generate [Fe_4_S_4_] clusters in the cytosol and nucleus. In the considered model, component CIA refers to all iron emanating from the labile iron pool (FC = LFeP) that is not imported into mitochondria or vacuoles. Since the nucleus, endoplasmic reticulum or other organelles are not included in the model, they have been implicitly incorporated into the cytosol. In a recent bioinformatics study [43], the cytosol, nucleus, and endoplasmic reticulum were predicted to contain 60, 30, and 460 µM Fe, respectively, based on fractional volumes of 0.615, 0.125, and 0.015, respectively. The total cellular concentration due to these three compartments would then be

$$[Fe_{ }]_{cia(cell)}=0.615[60]+0.125[30]+0.015[460]=47.55$$

If all of this iron were located in the cytosol, as assumed by this model, the [CIA] should equal 47.55/0.8 ≈ 60 µM. Consistent with that, the fractional volume of the “cytosol” in the model (0.8) is approximately the sum of the just-mentioned fractional volumes of the three compartments that are combined in the model. The values in the table are similar, including 80, 70, and 68 µM for W, Y, and D states. We assumed that the CIA concentration did not vary majorly between these cellular states since CIA iron (especially that in the nucleus) is thought to be fairly critical for cell viability and thus constant for all three cellular states.

***The labile iron pool (LFeP) concentration ([FC]):*** The LFeP is thought to arise from non-proteinaceous low-molecular Fe^II^ complexes in the cytosol. The size of this pool has received much attention and is not known with certainty. Our recent bioinformatic study [43] suggests a pool size of 10 - 50 µM Fe in Fe-replete whole cells. Nguyen et al. [27] reported a cytosolic LFeP concentration of 50 - 66 µM. The equivalent pool in *E. coli* cytosol was ~ 10 µM and 80-200 µM; see [47]. However, Brawley et al. [47] discovered that a significant percentage of this iron may have arisen from the degradation of ISCs during cytosol isolation in which case the endogenous LFeP would be smaller. The size of the LFeP at least in *E. coli* is sensitive to the concentration of iron in the media, with higher concentrations observed with increasing media iron concentrations. All things considered, in Table 2 we assigned the concentration of [FC] in the W state to be 20 µM and that in the D state to be 5 µM.

The remaining issue was what to assign [FC] for the Y state. Prior to the study of Chen et al. [48], the LFeP concentration in cytosol of Yfh1-deficient yeast cells was thought be diminished relative to in healthy cells (since the iron-regulation, known to be activated under Fe-deficient conditions, is also activated under Yfh1-deficient conditions). However, Chen et al. reported that the LFeP concentration in Yfh1-deficient cytosol was ***increased*** relative to in WT cells. This was (and remains) counterintuitive since the Aft1-iron regulon is activated in the Y state, indicating that frataxin-deficient cells “feel” iron-deficient. This conclusion remains controversial, and as discussed by Martelli and Puccio [49], mammalian cells with a frataxin deficiency are indeed iron-deficient. Whitnall et al. [50] found that the size of the LFeP in frataxin-deficient mice was diminished relative to healthy mice. For these reasons, we selected [FC] = 10 µM Fe for the Y state as it is midway between the values assumed for W and D states.

**Fig A. Example of a CRM that failed the Targeting filter.** This filter evaluates the accuracy of the steady state concentrations predicted with regulation, relative to those obtained without autoregulation. The plot below shows a transition between W without regulation →W with a CRM case added. This case was eliminated because it failed to generate accurate W steady state concentrations after the CRM was applied. The bottom plots were similar except that CRM case 1 was used. This case survived the filter because it was unperturbed (remained in the W state) when the CRM was included.


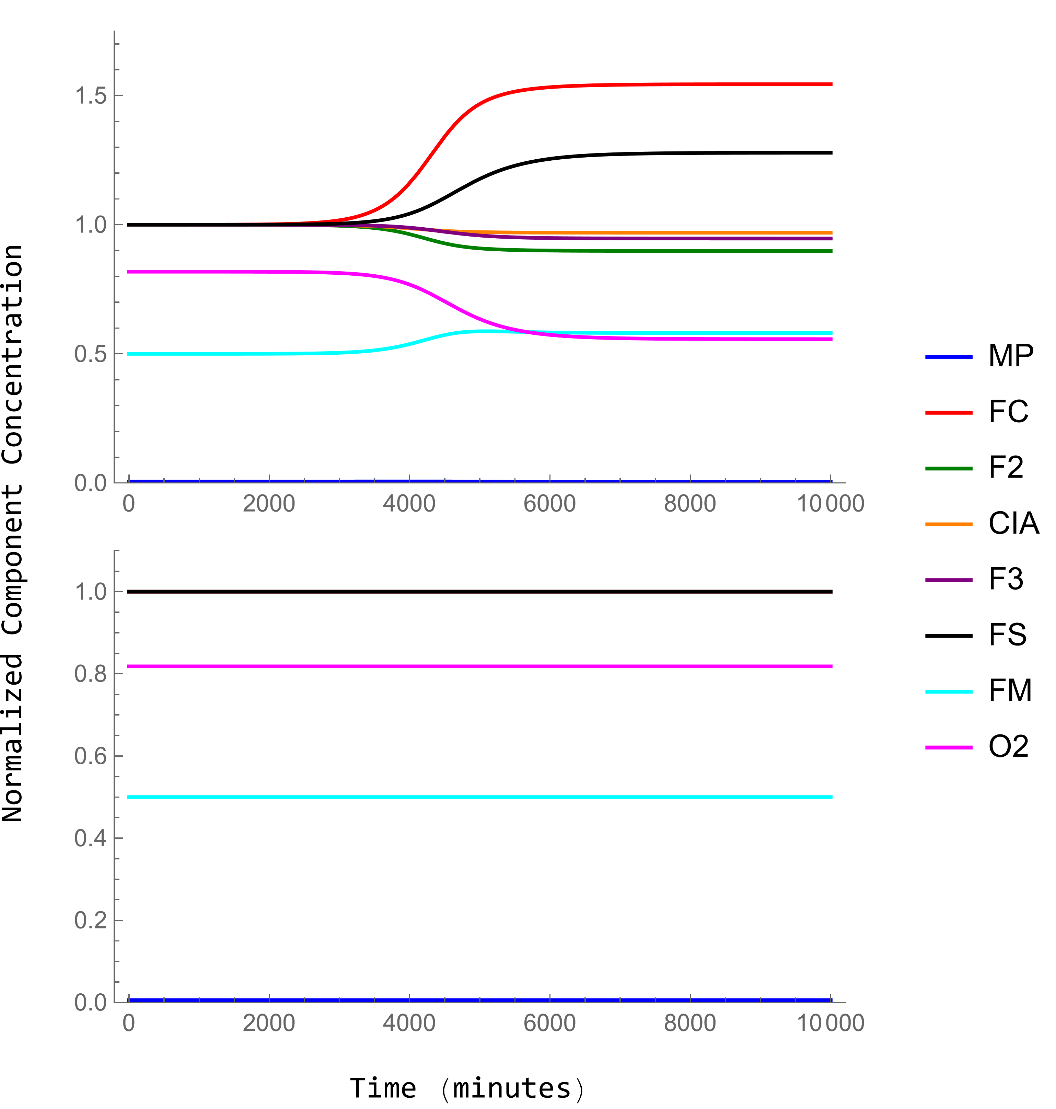


**Fig B. Example of a CRM that failed the Wandering filter.** This filter measures how much wandering occurred for a particular CRM case. For this case, the O2 plot for W → D wanders significantly compared to a straight line. The overall arc length was much longer than that of a straight line connecting the points, which is why this case was excluded.


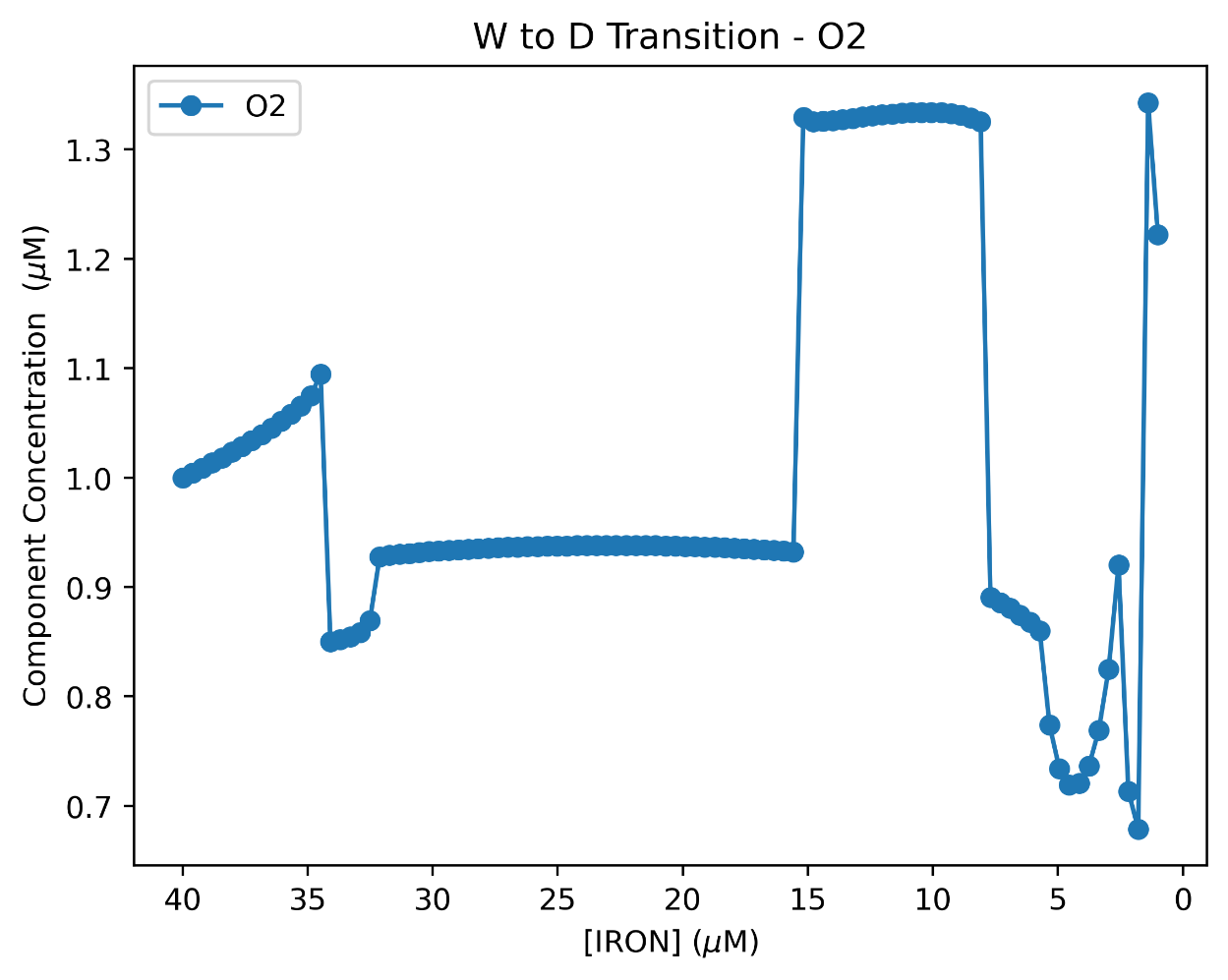


**Fig C. Example of a CRM that failed the Smoothness filter.** This filter measures a normalized distance between individual points for the steady state transition. The plot below does not involve much wandering away from the linear path, but there are many jumps or spikes between individual points towards the end. This CRM was eliminated (by the cluster method) for that reason; its smoothness score was substantially larger compared to the top 2 CRM cases.


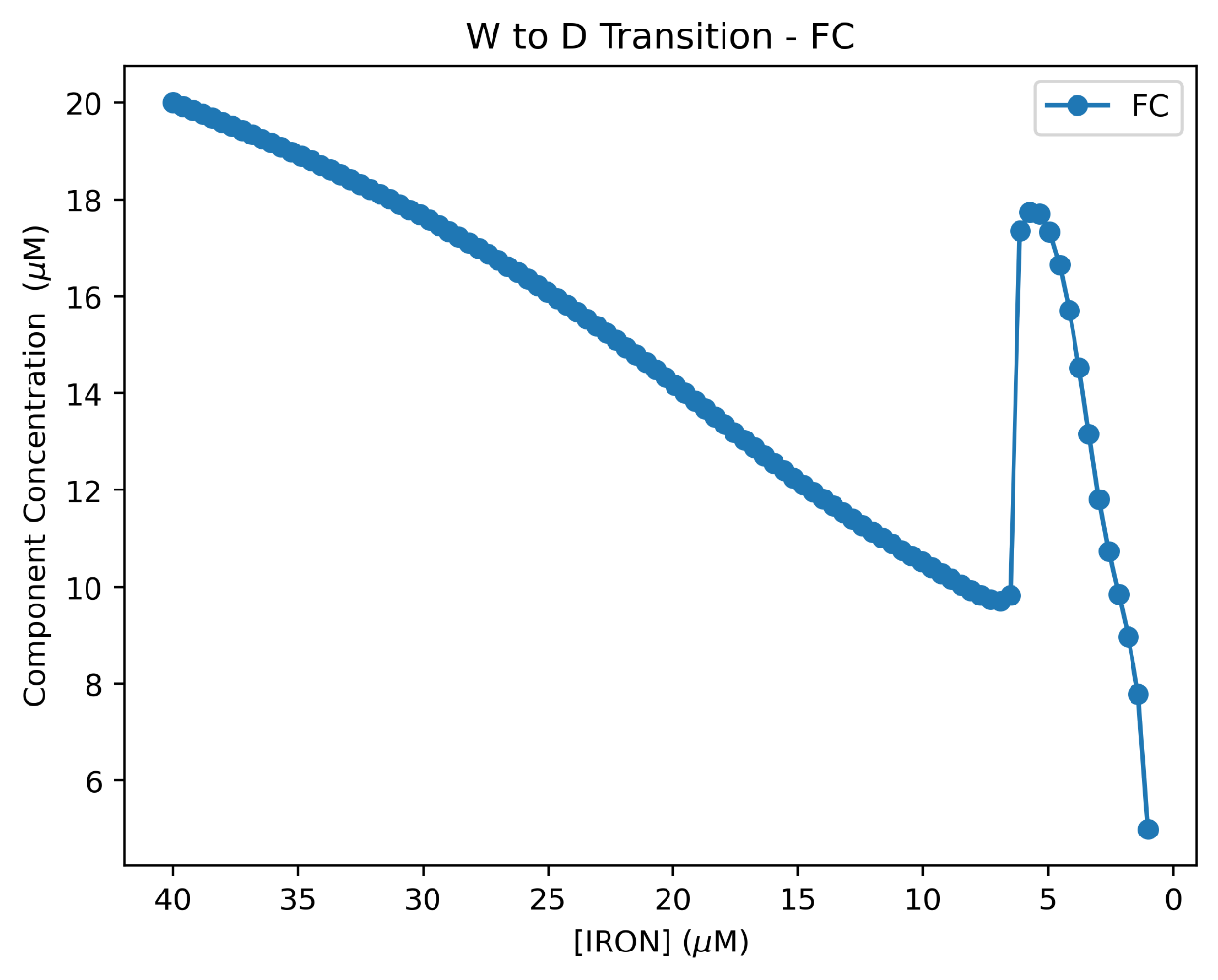

Supplement: S1 Text — [21, 33, 43–48]. Appendix A. How Rres = 9090 μM/min was selected; Appendix B. Jacobian matrices for the W, Y, and D states; Appendix C, Concentration estimates in Table 2; Fig A., example of a CRM that failed the Targeting filter; Fig B., example of a CRM that failed the Wandering filter; Fig C., example of a CRM that failed the Smoothness filter. (DOCX) [file pcbi.1011701.s001.docx]
